# Supplementary material for: Survival Outcomes of Immune Checkpoint Inhibitors in Conjunction with Cranial Radiation for Older Adults with Non-Small Cell Lung Cancer and Synchronous Brain Metastasis
Source: Curr Oncol. 2025 Sep 5;32(9):499. doi: 10.3390/curroncol32090499 (PMC12468429; doi:10.3390/curroncol32090499)
Supplement: Supplementary file 1 [file curroncol-32-00499-s001.zip › File S1-curroncol-3723038-supplementary methods TriNetX 0-15 days vs 30+ days.pdf]

TriNetX Explore real world, real-time global data

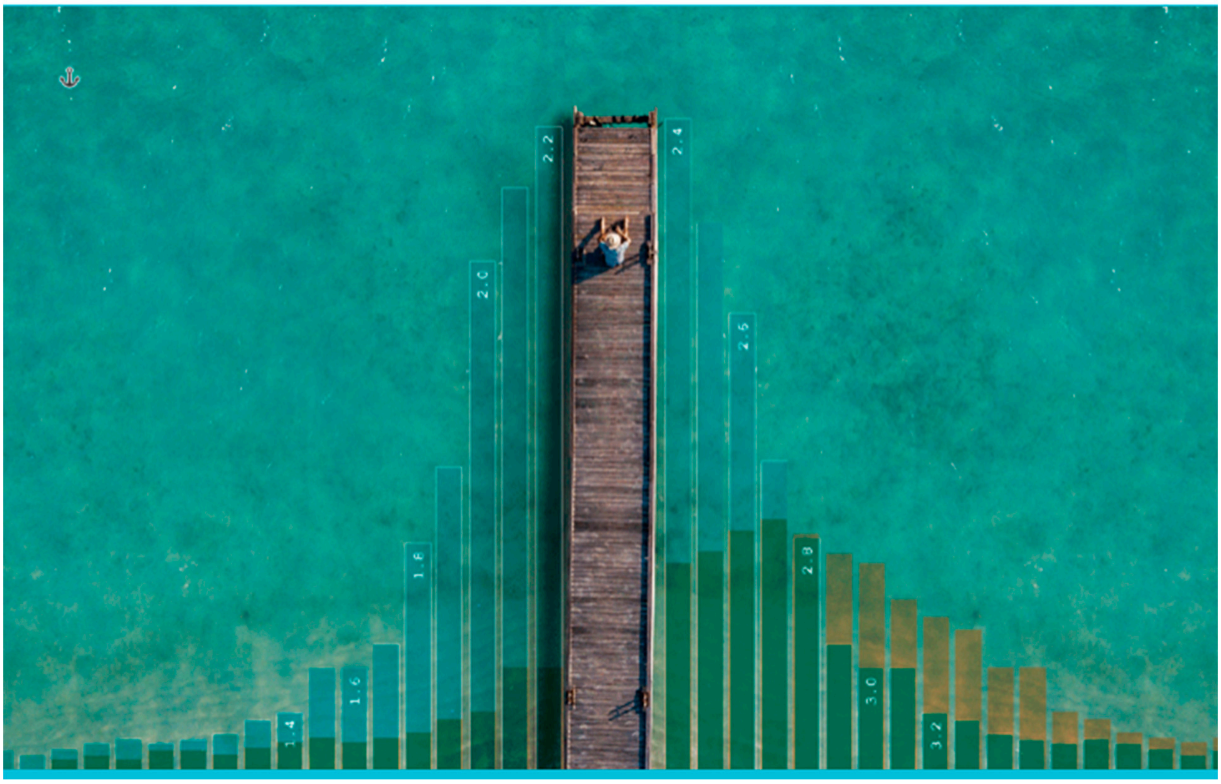

ICI+CR Survival

Compare Outcomes Analysis

Created by TriNetX on Aug 19, 2025, 16:40:33 UTC

Introduction

TriNetX is the global federated health research network providing access to electronic medical records (diagnoses, procedures, medications, laboratory values, genomic information) across large healthcare organizations (HCOs). This report was run on the set of HCOs grouped into a network called Global Collaborative Network. This network included 146 HCO(s).

This report describes a Compare Outcomes Analysis, named Compare grp 1 and 3 new, generated by the TriNetX platform on Aug 19, 2025, 16:40:33 UTC. This analysis compared the outcomes of two cohorts: Cohort A (2,014 patients) named ICI-CR 0-15 days and Cohort B (3,862 patients) named ICI-CR 31+days new.

This analysis was run by Ruchira Mahashabde (rvmahashabde@uams.edu) and downloaded by Ruchira Mahashabde (rvmahashabde@uams.edu).

Methods

The analysis process includes two main steps: 1) Defining the cohorts through query criteria; 2) Setting up and running the analysis. Setting up the analysis requires definitions for the index event, outcomes criteria, and the time frame. Compare outcomes supports four analyses: Measures of Association, Survival, Number of Instances and Lab result distribution. These analyses have additional options that are listed in the Outcomes Definitions and Analyses Specifications section below. Furthermore, characteristics of the cohorts that are balanced using propensity score matching are also included in the Propensity Score Matching section.

Cohorts definition

This section lists all terms used in the definitions of the two cohorts.

Query Criteria for Cohort 1 (query name: ICI-CR 0-15 days)

This query was run on the network Global Collaborative Network with 146 HCO(s) queried and 146 HCO(s) responded. A total of 29 provider(s) responded with patients. The final cohort included 2,014 patients who matched the query criteria listed in the table below. For the text representation of the query criteria please see Appendix A.

**Ungrouped terms**				
must have		demographics	Age	Age (between 65 and 120 years (most recent occurrence))
cannot have		medication	NLM:RXNORM:1535457	ceritinib
	or	medication	NLM:RXNORM:1430438	afatinib
	or	medication	NLM:RXNORM:1727455	alectinib
	or	medication	NLM:RXNORM:1148495	crizotinib
	or	medication	NLM:RXNORM:1921217	brigatinib
	or	medication	NLM:RXNORM:337525	erlotinib
	or	medication	NLM:RXNORM:1424911	dabrafenib
	or	medication	NLM:RXNORM:328134	gefitinib
	or	medication	NLM:RXNORM:1721560	osimertinib
	or	medication	NLM:RXNORM:1425099	trametinib
**Group 1**				
**Group 1A NSCLC DX**				

must have	any of	diagnosis	UMLS:ICD10CM:C34	Malignant neoplasm of bronchus and lung
		globaloncology	UMLS:ICDO3:C34	Bronchus and lung
		diagnosis	UMLS:ICD10CM:C34	Malignant neoplasm of bronchus and lung
		globaloncology	TNX:STAGE:S1	Stage 1
		globaloncology	TNX:STAGE:S2	Stage 2
		globaloncology	TNX:STAGE:S3	Stage 3
		globaloncology	TNX:TNX:NSCC	Non-small cell carcinoma
		diagnosis	UMLS:ICD10CM:C34	Malignant neoplasm of bronchus and lung
		genomic	GENE:17635	CD274
		laboratory	UMLS:LNC:83053-9	Cells.programmed cell death ligand 1/100 viable tumor cells in Tissue by Immune stain
cannot have		globaloncology	TNX:TNX:SCC	Small cell carcinoma
	or	globaloncology	UMLS:ICDO3:8041/3	Small cell carcinoma, NOS
	or	globaloncology	UMLS:ICDO3:8045/3	Combined small cell carcinoma
	or	globaloncology	UMLS:ICDO3:8043/3	Small cell carcinoma, fusiform cell
	or	globaloncology	UMLS:ICDO3:8044/3	Small cell carcinoma, intermediate cell
	or	genomic	GENE:3236	EGFR
	or	genomic	GENE:427	ALK
date constraint		The terms in this group occurred at any time		
event relationship		Any instance of BM DX occurred within 1 day before or up to 1 day after any instance of NSCLC DX		
**Group 1B BM DX**				
must have	any of	diagnosis	UMLS:ICD10CM:C79.31	Secondary malignant neoplasm of brain
		globaloncology	TNX:STAGE:S4	Stage 4
		globaloncology	TNX:STAGE:TNM_M1	M1
**Group 2**				
**Group 2A ICI**				
must have	any of	procedure	UMLS:HCPCS:J9271	Injection, pembrolizumab, 1 mg
		procedure	UMLS:HCPCS:C9027	Injection, pembrolizumab, 1 mg (deprecated 2015)
		procedure	UMLS:HCPCS:J9299	Injection, nivolumab, 1 mg
		procedure	UMLS:HCPCS:J9298	Injection, nivolumab and relatlimab-rmbw, 3 mg/1 mg
		procedure	UMLS:HCPCS:C9453	Injection, nivolumab, 1 mg (deprecated 2015)
		procedure	UMLS:HCPCS:J9228	Injection, ipilimumab, 1 mg
		procedure	UMLS:HCPCS:J9022	Injection, atezolizumab, 10 mg
		procedure	UMLS:HCPCS:C9483	Injection, atezolizumab, 10 mg (deprecated 2017)
		procedure	UMLS:HCPCS:J9023	Injection, avelumab, 10 mg
		procedure	UMLS:HCPCS:C9491	Injection, avelumab, 10 mg (deprecated 2017)
		procedure	UMLS:HCPCS:J9173	Injection, durvalumab, 10 mg
		procedure	UMLS:HCPCS:J9119	Injection, cemiplimab-rwlc, 1 mg
		procedure	UMLS:HCPCS:C9492	Injection, durvalumab, 10 mg (deprecated 2018)
		procedure	UMLS:HCPCS:C9044	Injection, cemiplimab-rwlc, 1 mg (deprecated 2019)
cannot have		medication	NLM:RXNORM:1535457	ceritinib
	or	medication	NLM:RXNORM:1430438	afatinib
	or	medication	NLM:RXNORM:1727455	alectinib
	or	medication	NLM:RXNORM:1148495	crizotinib
	or	medication	NLM:RXNORM:1921217	brigatinib
	or	medication	NLM:RXNORM:337525	erlotinib

	or	medication	NLM:RXNORM:1424911	dabrafenib
	or	medication	NLM:RXNORM:328134	gefitinib
	or	medication	NLM:RXNORM:1721560	osimertinib
	or	medication	NLM:RXNORM:1425099	trametinib
date constraint		The terms in this group occurred at any time		
event relationship		Any instance of NSCLC DX occurred within 6 months on or before any instance of ICI		
**Group 2B NSCLC DX**				
must have	any of	diagnosis	UMLS:ICD10CM:C34	Malignant neoplasm of bronchus and lung
		globaloncology	UMLS:ICDO3:C34	Bronchus and lung
		diagnosis	UMLS:ICD10CM:C34	Malignant neoplasm of bronchus and lung
		globaloncology	TNX:STAGE:S1	Stage 1
		globaloncology	TNX:STAGE:S2	Stage 2
		globaloncology	TNX:STAGE:S3	Stage 3
		globaloncology	TNX:TNX:NSCC	Non-small cell carcinoma
		diagnosis	UMLS:ICD10CM:C34	Malignant neoplasm of bronchus and lung
		genomic	GENE:17635	CD274
		laboratory	UMLS:LNC:83053-9	Cells.programmed cell death ligand 1/100 viable tumor cells in Tissue by Immune stain
cannot have		globaloncology	TNX:TNX:SCC	Small cell carcinoma
	or	globaloncology	UMLS:ICDO3:8041/3	Small cell carcinoma, NOS
	or	globaloncology	UMLS:ICDO3:8045/3	Combined small cell carcinoma
	or	globaloncology	UMLS:ICDO3:8043/3	Small cell carcinoma, fusiform cell
	or	globaloncology	UMLS:ICDO3:8044/3	Small cell carcinoma, intermediate cell
	or	genomic	GENE:3236	EGFR
	or	genomic	GENE:427	ALK
**Group 3**				
**Group 3A CR**				
must have	any of	procedure	UMLS:CPT:61796	Stereotactic radiosurgery (particle beam, gamma ray, or linear accelerator); 1 simple cranial lesion
		procedure	UMLS:CPT:61798	Stereotactic radiosurgery (particle beam, gamma ray, or linear accelerator); 1 complex cranial lesion
		procedure	UMLS:CPT:61799	Stereotactic radiosurgery (particle beam, gamma ray, or linear accelerator); each additional cranial lesion, complex (List separately in addition to code for primary procedure)
		procedure	UMLS:CPT:61800	Application of stereotactic headframe for stereotactic radiosurgery (List separately in addition to code for primary procedure)
		procedure	UMLS:CPT:77371	Radiation treatment delivery, stereotactic radiosurgery (SRS), complete course of treatment of cranial lesion(s) consisting of 1 session; multi-source Cobalt 60 based
		procedure	UMLS:CPT:77372	Radiation treatment delivery, stereotactic radiosurgery (SRS), complete course of treatment of cranial lesion(s) consisting of 1 session; linear accelerator based
		procedure	UMLS:CPT:77432	Stereotactic radiation treatment management of cranial lesion(s)

		(complete course of treatment consisting of 1 session)
procedure	UMLS:HCPCS:G0339	Image-guided robotic linear accelerator-based stereotactic radiosurgery, complete course of therapy in one session or first session of fractionated treatment
procedure	UMLS:HCPCS:G0340	Image-guided robotic linear accelerator-based stereotactic radiosurgery, delivery including collimator changes and custom plugging, fractionated treatment, all lesions, per session, second through fifth sessions, maximum five sessions per course of treatment
procedure	UMLS:CPT:77401	Radiation treatment delivery, superficial and/or ortho voltage, per day
procedure	UMLS:CPT:77402	Radiation treatment delivery, >=1 MeV; simple
procedure	UMLS:CPT:77407	Radiation treatment delivery, >=1 MeV; intermediate
procedure	UMLS:CPT:77412	Radiation treatment delivery, >=1 MeV; complex
procedure	UMLS:HCPCS:G6004	Radiation treatment delivery, single treatment area,single port or parallel opposed ports, simple blocks or no blocks: 6-10 mev
procedure	UMLS:HCPCS:G6005	Radiation treatment delivery, single treatment area,single port or parallel opposed ports, simple blocks or no blocks: 11-19 mev
procedure	UMLS:HCPCS:G6006	Radiation treatment delivery, single treatment area,single port or parallel opposed ports, simple blocks or no blocks: 20 mev or greater
procedure	UMLS:HCPCS:G6007	Radiation treatment delivery, 2 separate treatment areas, 3 or more ports on a single treatment area, use of multiple blocks: up to 5 mev
procedure	UMLS:HCPCS:G6008	Radiation treatment delivery, 2 separate treatment areas, 3 or more ports on a single treatment area, use of multiple blocks: 6-10 mev
procedure	UMLS:HCPCS:G6009	Radiation treatment delivery, 2 separate treatment areas, 3 or more ports on a single treatment area, use of multiple blocks: 11-19 mev
procedure	UMLS:HCPCS:G6010	Radiation treatment delivery, 2 separate treatment areas, 3 or more ports on a single treatment area, use of multiple blocks: 20 mev or greater
procedure	UMLS:HCPCS:G6011	Radiation treatment delivery,3 or more separate treatment areas, custom blocking, tangential ports, wedges, rotational beam, compensators, electron beam; up to 5 mev
procedure	UMLS:HCPCS:G6012	Radiation treatment delivery,3 or more separate treatment areas, custom

				blocking, tangential ports, wedges, rotational beam, compensators, electron beam; 6-10 mev
		procedure	UMLS:HCPCS:G6013	Radiation treatment delivery,3 or more separate treatment areas, custom blocking, tangential ports, wedges, rotational beam, compensators, electron beam; 11-19 mev
		procedure	UMLS:HCPCS:G6014	Radiation treatment delivery,3 or more separate treatment areas, custom blocking, tangential ports, wedges, rotational beam, compensators, electron beam; 20 mev or greater
		procedure	UMLS:HCPCS:G6016	Compensator-based beam modulation treatment delivery of inverse planned treatment using 3 or more high resolution (milled or cast) compensator, convergent beam modulated fields, per treatment session
		procedure	UMLS:CPT:77373	Stereotactic body radiation therapy, treatment delivery, per fraction to 1 or more lesions, including image guidance, entire course not to exceed 5 fractions
date constraint		The terms in this group occurred at any time		
event relationship		Any instance of NSCLC DX occurred within 6 months on or before any instance of CR		
**Group 3B NSCLC DX**				
must have	any of	diagnosis	UMLS:ICD10CM:C34	Malignant neoplasm of bronchus and lung
		globaloncology	UMLS:ICDO3:C34	Bronchus and lung
		diagnosis	UMLS:ICD10CM:C34	Malignant neoplasm of bronchus and lung
		globaloncology	TNX:STAGE:S1	Stage 1
		globaloncology	TNX:STAGE:S2	Stage 2
		globaloncology	TNX:STAGE:S3	Stage 3
		globaloncology	TNX:TNX:NSCC	Non-small cell carcinoma
		diagnosis	UMLS:ICD10CM:C34	Malignant neoplasm of bronchus and lung
		genomic	GENE:17635	CD274
		laboratory	UMLS:LNC:83053-9	Cells.programmed cell death ligand 1/100 viable tumor cells in Tissue by Immune stain
cannot have		globaloncology	TNX:TNX:SCC	Small cell carcinoma
	or	globaloncology	UMLS:ICDO3:8041/3	Small cell carcinoma, NOS
	or	globaloncology	UMLS:ICDO3:8045/3	Combined small cell carcinoma
	or	globaloncology	UMLS:ICDO3:8043/3	Small cell carcinoma, fusiform cell
	or	globaloncology	UMLS:ICDO3:8044/3	Small cell carcinoma, intermediate cell
	or	genomic	GENE:3236	EGFR
	or	genomic	GENE:427	ALK
**Group 4**				
**Group 4A ICI**				
must have	any of	procedure	UMLS:HCPCS:J9271	Injection, pembrolizumab, 1 mg
		procedure	UMLS:HCPCS:C9027	Injection, pembrolizumab, 1 mg (deprecated 2015)
		procedure	UMLS:HCPCS:J9299	Injection, nivolumab, 1 mg
		procedure	UMLS:HCPCS:J9298	Injection, nivolumab and relatlimab-rmbw, 3 mg/1 mg
		procedure	UMLS:HCPCS:C9453	Injection, nivolumab, 1 mg (deprecated 2015)

		procedure	UMLS:HCPCS:J9228	Injection, ipilimumab, 1 mg
		procedure	UMLS:HCPCS:J9022	Injection, atezolizumab, 10 mg
		procedure	UMLS:HCPCS:C9483	Injection, atezolizumab, 10 mg (deprecated 2017)
		procedure	UMLS:HCPCS:J9023	Injection, avelumab, 10 mg
		procedure	UMLS:HCPCS:C9491	Injection, avelumab, 10 mg (deprecated 2017)
		procedure	UMLS:HCPCS:J9173	Injection, durvalumab, 10 mg
		procedure	UMLS:HCPCS:J9119	Injection, cemiplimab-rwlc, 1 mg
		procedure	UMLS:HCPCS:C9492	Injection, durvalumab, 10 mg (deprecated 2018)
		procedure	UMLS:HCPCS:C9044	Injection, cemiplimab-rwlc, 1 mg (deprecated 2019)
date constraint		The terms in this group occurred at any time		
event relationship		Any instance of CR occurred within 15 days before or up to 15 days after any instance of ICI		
**Group 4B CR**				
must have	any of	procedure	UMLS:CPT:61796	Stereotactic radiosurgery (particle beam, gamma ray, or linear accelerator); 1 simple cranial lesion
		procedure	UMLS:CPT:61798	Stereotactic radiosurgery (particle beam, gamma ray, or linear accelerator); 1 complex cranial lesion
		procedure	UMLS:CPT:61799	Stereotactic radiosurgery (particle beam, gamma ray, or linear accelerator); each additional cranial lesion, complex (List separately in addition to code for primary procedure)
		procedure	UMLS:CPT:61800	Application of stereotactic headframe for stereotactic radiosurgery (List separately in addition to code for primary procedure)
		procedure	UMLS:CPT:77371	Radiation treatment delivery, stereotactic radiosurgery (SRS), complete course of treatment of cranial lesion(s) consisting of 1 session; multi-source Cobalt 60 based
		procedure	UMLS:CPT:77372	Radiation treatment delivery, stereotactic radiosurgery (SRS), complete course of treatment of cranial lesion(s) consisting of 1 session; linear accelerator based
		procedure	UMLS:CPT:77432	Stereotactic radiation treatment management of cranial lesion(s) (complete course of treatment consisting of 1 session)
		procedure	UMLS:HCPCS:G0339	Image-guided robotic linear accelerator-based stereotactic radiosurgery, complete course of therapy in one session or first session of fractionated treatment
		procedure	UMLS:HCPCS:G0340	Image-guided robotic linear accelerator-based stereotactic radiosurgery, delivery including collimator changes and custom plugging, fractionated treatment, all lesions, per session, second through fifth sessions,

		maximum five sessions per course of treatment
procedure	UMLS:CPT:77401	Radiation treatment delivery, superficial and/or ortho voltage, per day
procedure	UMLS:CPT:77402	Radiation treatment delivery, >=1 MeV; simple
procedure	UMLS:CPT:77407	Radiation treatment delivery, >=1 MeV; intermediate
procedure	UMLS:CPT:77412	Radiation treatment delivery, >=1 MeV; complex
procedure	UMLS:HCPCS:G6004	Radiation treatment delivery, single treatment area,single port or parallel opposed ports, simple blocks or no blocks: 6-10 mev
procedure	UMLS:HCPCS:G6005	Radiation treatment delivery, single treatment area,single port or parallel opposed ports, simple blocks or no blocks: 11-19 mev
procedure	UMLS:HCPCS:G6006	Radiation treatment delivery, single treatment area,single port or parallel opposed ports, simple blocks or no blocks: 20 mev or greater
procedure	UMLS:HCPCS:G6007	Radiation treatment delivery, 2 separate treatment areas, 3 or more ports on a single treatment area, use of multiple blocks: up to 5 mev
procedure	UMLS:HCPCS:G6008	Radiation treatment delivery, 2 separate treatment areas, 3 or more ports on a single treatment area, use of multiple blocks: 6-10 mev
procedure	UMLS:HCPCS:G6009	Radiation treatment delivery, 2 separate treatment areas, 3 or more ports on a single treatment area, use of multiple blocks: 11-19 mev
procedure	UMLS:HCPCS:G6010	Radiation treatment delivery, 2 separate treatment areas, 3 or more ports on a single treatment area, use of multiple blocks: 20 mev or greater
procedure	UMLS:HCPCS:G6011	Radiation treatment delivery,3 or more separate treatment areas, custom blocking, tangential ports, wedges, rotational beam, compensators, electron beam; up to 5 mev
procedure	UMLS:HCPCS:G6012	Radiation treatment delivery,3 or more separate treatment areas, custom blocking, tangential ports, wedges, rotational beam, compensators, electron beam; 6-10 mev
procedure	UMLS:HCPCS:G6013	Radiation treatment delivery,3 or more separate treatment areas, custom blocking, tangential ports, wedges, rotational beam, compensators, electron beam; 11-19 mev
procedure	UMLS:HCPCS:G6014	Radiation treatment delivery,3 or more separate treatment areas, custom blocking, tangential ports, wedges, rotational beam, compensators, electron beam; 20 mev or greater

		procedure	UMLS:HCPCS:G6016	Compensator-based beam modulation treatment delivery of inverse planned treatment using 3 or more high resolution (milled or cast) compensator, convergent beam modulated fields, per treatment session
		procedure	UMLS:CPT:77373	Stereotactic body radiation therapy, treatment delivery, per fraction to 1 or more lesions, including image guidance, entire course not to exceed 5 fractions

Query Criteria for Cohort 2 (query name: ICI-CR 31+days new)

This query was run on the network Global Collaborative Network with 154 HCO(s) queried and 154 HCO(s) responded. A total of 34 provider(s) responded with patients. The final cohort included 3,862 patients who matched the query criteria listed in the table below.

**Ungrouped terms**				
must have		demographics	Age	Age (between 65 and 120 years (most recent occurrence))
**Group 1**				
**Group 1A NSCLC DX**				
must have	any of	diagnosis	UMLS:ICD10CM:C34	Malignant neoplasm of bronchus and lung
		globaloncology	UMLS:ICDO3:C34	Bronchus and lung
		diagnosis	UMLS:ICD10CM:C34	Malignant neoplasm of bronchus and lung
		globaloncology	TNX:STAGE:S1	Stage 1
		globaloncology	TNX:STAGE:S2	Stage 2
		globaloncology	TNX:STAGE:S3	Stage 3
		globaloncology	TNX:TNX:NSCC	Non-small cell carcinoma
		diagnosis	UMLS:ICD10CM:C34	Malignant neoplasm of bronchus and lung
		genomic	GENE:17635	CD274
		laboratory	UMLS:LNC:83053-9	Cells.programmed cell death ligand 1/100 viable tumor cells in Tissue by Immune stain
cannot have		globaloncology	TNX:TNX:SCC	Small cell carcinoma
	or	globaloncology	UMLS:ICDO3:8041/3	Small cell carcinoma, NOS
	or	globaloncology	UMLS:ICDO3:8045/3	Combined small cell carcinoma
	or	globaloncology	UMLS:ICDO3:8043/3	Small cell carcinoma, fusiform cell
	or	globaloncology	UMLS:ICDO3:8044/3	Small cell carcinoma, intermediate cell
	or	genomic	GENE:3236	EGFR
	or	genomic	GENE:427	ALK
date constraint		The terms in this group occurred at any time		
event relationship		Any instance of BM DX occurred within 1 day before or up to 1 day after any instance of NSCLC DX		
**Group 1B BM DX**				
must have	any of	diagnosis	UMLS:ICD10CM:C79.31	Secondary malignant neoplasm of brain
		globaloncology	TNX:STAGE:S4	Stage 4
		globaloncology	TNX:STAGE:TNM_M1	M1
**Group 2**				
**Group 2A ICI**				
must have	any of	procedure	UMLS:HCPCS:J9271	Injection, pembrolizumab, 1 mg
		procedure	UMLS:HCPCS:C9027	Injection, pembrolizumab, 1 mg (deprecated 2015)
		procedure	UMLS:HCPCS:J9299	Injection, nivolumab, 1 mg

		procedure	UMLS:HCPCS:J9298	Injection, nivolumab and relatlimab-rmbw, 3 mg/1 mg
		procedure	UMLS:HCPCS:C9453	Injection, nivolumab, 1 mg (deprecated 2015)
		procedure	UMLS:HCPCS:J9228	Injection, ipilimumab, 1 mg
		procedure	UMLS:HCPCS:J9022	Injection, atezolizumab, 10 mg
		procedure	UMLS:HCPCS:C9483	Injection, atezolizumab, 10 mg (deprecated 2017)
		procedure	UMLS:HCPCS:J9023	Injection, avelumab, 10 mg
		procedure	UMLS:HCPCS:C9491	Injection, avelumab, 10 mg (deprecated 2017)
		procedure	UMLS:HCPCS:J9173	Injection, durvalumab, 10 mg
		procedure	UMLS:HCPCS:J9119	Injection, cemiplimab-rwlc, 1 mg
		procedure	UMLS:HCPCS:C9492	Injection, durvalumab, 10 mg (deprecated 2018)
		procedure	UMLS:HCPCS:C9044	Injection, cemiplimab-rwlc, 1 mg (deprecated 2019)
cannot have		medication	NLM:RXNORM:1535457	ceritinib
	or	medication	NLM:RXNORM:1430438	afatinib
	or	medication	NLM:RXNORM:1727455	alectinib
	or	medication	NLM:RXNORM:1148495	crizotinib
	or	medication	NLM:RXNORM:1921217	brigatinib
	or	medication	NLM:RXNORM:337525	erlotinib
	or	medication	NLM:RXNORM:1424911	dabrafenib
	or	medication	NLM:RXNORM:328134	gefitinib
	or	medication	NLM:RXNORM:1721560	osimertinib
	or	medication	NLM:RXNORM:1425099	trametinib
date constraint		The terms in this group occurred at any time		
event relationship		Any instance of NSCLC DX occurred within 6 months on or before any instance of ICI		
**Group 2B NSCLC DX**				
must have	any of	diagnosis	UMLS:ICD10CM:C34	Malignant neoplasm of bronchus and lung
		globaloncology	UMLS:ICDO3:C34	Bronchus and lung
		diagnosis	UMLS:ICD10CM:C34	Malignant neoplasm of bronchus and lung
		globaloncology	TNX:STAGE:S1	Stage 1
		globaloncology	TNX:STAGE:S2	Stage 2
		globaloncology	TNX:STAGE:S3	Stage 3
		globaloncology	TNX:TNX:NSCC	Non-small cell carcinoma
		diagnosis	UMLS:ICD10CM:C34	Malignant neoplasm of bronchus and lung
		genomic	GENE:17635	CD274
		laboratory	UMLS:LNC:83053-9	Cells.programmed cell death ligand 1/100 viable tumor cells in Tissue by Immune stain
cannot have		globaloncology	TNX:TNX:SCC	Small cell carcinoma
	or	globaloncology	UMLS:ICDO3:8041/3	Small cell carcinoma, NOS
	or	globaloncology	UMLS:ICDO3:8045/3	Combined small cell carcinoma
	or	globaloncology	UMLS:ICDO3:8043/3	Small cell carcinoma, fusiform cell
	or	globaloncology	UMLS:ICDO3:8044/3	Small cell carcinoma, intermediate cell
	or	genomic	GENE:3236	EGFR
	or	genomic	GENE:427	ALK
**Group 3**				
**Group 3A CR**				
must have	any of	procedure	UMLS:CPT:61796	Stereotactic radiosurgery (particle beam, gamma ray, or linear accelerator); 1 simple cranial lesion

procedure	UMLS:CPT:61798	Stereotactic radiosurgery (particle beam, gamma ray, or linear accelerator); 1 complex cranial lesion
procedure	UMLS:CPT:61799	Stereotactic radiosurgery (particle beam, gamma ray, or linear accelerator); each additional cranial lesion, complex (List separately in addition to code for primary procedure)
procedure	UMLS:CPT:61800	Application of stereotactic headframe for stereotactic radiosurgery (List separately in addition to code for primary procedure)
procedure	UMLS:CPT:77371	Radiation treatment delivery, stereotactic radiosurgery (SRS), complete course of treatment of cranial lesion(s) consisting of 1 session; multi-source Cobalt 60 based
procedure	UMLS:CPT:77372	Radiation treatment delivery, stereotactic radiosurgery (SRS), complete course of treatment of cranial lesion(s) consisting of 1 session; linear accelerator based
procedure	UMLS:CPT:77432	Stereotactic radiation treatment management of cranial lesion(s) (complete course of treatment consisting of 1 session)
procedure	UMLS:HCPCS:G0339	Image-guided robotic linear accelerator-based stereotactic radiosurgery, complete course of therapy in one session or first session of fractionated treatment
procedure	UMLS:HCPCS:G0340	Image-guided robotic linear accelerator-based stereotactic radiosurgery, delivery including collimator changes and custom plugging, fractionated treatment, all lesions, per session, second through fifth sessions, maximum five sessions per course of treatment
procedure	UMLS:CPT:77401	Radiation treatment delivery, superficial and/or ortho voltage, per day
procedure	UMLS:CPT:77402	Radiation treatment delivery, >=1 MeV; simple
procedure	UMLS:CPT:77407	Radiation treatment delivery, >=1 MeV; intermediate
procedure	UMLS:CPT:77412	Radiation treatment delivery, >=1 MeV; complex
procedure	UMLS:HCPCS:G6004	Radiation treatment delivery, single treatment area,single port or parallel opposed ports, simple blocks or no blocks: 6-10 mev
procedure	UMLS:HCPCS:G6005	Radiation treatment delivery, single treatment area,single port or parallel opposed ports, simple blocks or no blocks: 11-19 mev
procedure	UMLS:HCPCS:G6006	Radiation treatment delivery, single treatment area,single port or parallel

				opposed ports, simple blocks or no blocks: 20 mev or greater
		procedure	UMLS:HCPCS:G6007	Radiation treatment delivery, 2 separate treatment areas, 3 or more ports on a single treatment area, use of multiple blocks: up to 5 mev
		procedure	UMLS:HCPCS:G6008	Radiation treatment delivery, 2 separate treatment areas, 3 or more ports on a single treatment area, use of multiple blocks: 6-10 mev
		procedure	UMLS:HCPCS:G6009	Radiation treatment delivery, 2 separate treatment areas, 3 or more ports on a single treatment area, use of multiple blocks: 11-19 mev
		procedure	UMLS:HCPCS:G6010	Radiation treatment delivery, 2 separate treatment areas, 3 or more ports on a single treatment area, use of multiple blocks: 20 mev or greater
		procedure	UMLS:HCPCS:G6011	Radiation treatment delivery,3 or more separate treatment areas, custom blocking, tangential ports, wedges, rotational beam, compensators, electron beam; up to 5 mev
		procedure	UMLS:HCPCS:G6012	Radiation treatment delivery,3 or more separate treatment areas, custom blocking, tangential ports, wedges, rotational beam, compensators, electron beam; 6-10 mev
		procedure	UMLS:HCPCS:G6013	Radiation treatment delivery,3 or more separate treatment areas, custom blocking, tangential ports, wedges, rotational beam, compensators, electron beam; 11-19 mev
		procedure	UMLS:HCPCS:G6014	Radiation treatment delivery,3 or more separate treatment areas, custom blocking, tangential ports, wedges, rotational beam, compensators, electron beam; 20 mev or greater
		procedure	UMLS:HCPCS:G6016	Compensator-based beam modulation treatment delivery of inverse planned treatment using 3 or more high resolution (milled or cast) compensator, convergent beam modulated fields, per treatment session
		procedure	UMLS:CPT:77373	Stereotactic body radiation therapy, treatment delivery, per fraction to 1 or more lesions, including image guidance, entire course not to exceed 5 fractions
date constraint		The terms in this group occurred at any time		
event relationship		Any instance of NSCLC DX occurred within 6 months on or before any instance of CR		
**Group 3B NSCLC DX**				
must have	any of	diagnosis	UMLS:ICD10CM:C34	Malignant neoplasm of bronchus and lung
		globaloncology	UMLS:ICDO3:C34	Bronchus and lung
		diagnosis	UMLS:ICD10CM:C34	Malignant neoplasm of bronchus and lung
		globaloncology	TNX:STAGE:S1	Stage 1
		globaloncology	TNX:STAGE:S2	Stage 2

		globaloncology	TNX:STAGE:S3	Stage 3
		globaloncology	TNX:TNX:NSCC	Non-small cell carcinoma
		diagnosis	UMLS:ICD10CM:C34	Malignant neoplasm of bronchus and lung
		genomic	GENE:17635	CD274
		laboratory	UMLS:LNC:83053-9	Cells.programmed cell death ligand 1/100 viable tumor cells in Tissue by Immune stain
cannot have		globaloncology	TNX:TNX:SCC	Small cell carcinoma
	or	globaloncology	UMLS:ICDO3:8041/3	Small cell carcinoma, NOS
	or	globaloncology	UMLS:ICDO3:8045/3	Combined small cell carcinoma
	or	globaloncology	UMLS:ICDO3:8043/3	Small cell carcinoma, fusiform cell
	or	globaloncology	UMLS:ICDO3:8044/3	Small cell carcinoma, intermediate cell
	or	genomic	GENE:3236	EGFR
	or	genomic	GENE:427	ALK
**Group 4 or Group 5 must be present**				
**Group 4**				
**Group 4A ICI**				
must have	any of	procedure	UMLS:HCPCS:J9271	Injection, pembrolizumab, 1 mg
		procedure	UMLS:HCPCS:C9027	Injection, pembrolizumab, 1 mg (deprecated 2015)
		procedure	UMLS:HCPCS:J9299	Injection, nivolumab, 1 mg
		procedure	UMLS:HCPCS:J9298	Injection, nivolumab and relatlimab-rmbw, 3 mg/1 mg
		procedure	UMLS:HCPCS:C9453	Injection, nivolumab, 1 mg (deprecated 2015)
		procedure	UMLS:HCPCS:J9228	Injection, ipilimumab, 1 mg
		procedure	UMLS:HCPCS:J9022	Injection, atezolizumab, 10 mg
		procedure	UMLS:HCPCS:C9483	Injection, atezolizumab, 10 mg (deprecated 2017)
		procedure	UMLS:HCPCS:J9023	Injection, avelumab, 10 mg
		procedure	UMLS:HCPCS:C9491	Injection, avelumab, 10 mg (deprecated 2017)
		procedure	UMLS:HCPCS:J9173	Injection, durvalumab, 10 mg
		procedure	UMLS:HCPCS:J9119	Injection, cemiplimab-rwlc, 1 mg
		procedure	UMLS:HCPCS:C9492	Injection, durvalumab, 10 mg (deprecated 2018)
		procedure	UMLS:HCPCS:C9044	Injection, cemiplimab-rwlc, 1 mg (deprecated 2019)
date constraint		The terms in this group occurred at any time		
event relationship		Any instance of CR occurred within 31 days and 6 months after any instance of ICI		
**Group 4B CR**				
must have	any of	procedure	UMLS:CPT:61796	Stereotactic radiosurgery (particle beam, gamma ray, or linear accelerator); 1 simple cranial lesion
		procedure	UMLS:CPT:61798	Stereotactic radiosurgery (particle beam, gamma ray, or linear accelerator); 1 complex cranial lesion
		procedure	UMLS:CPT:61799	Stereotactic radiosurgery (particle beam, gamma ray, or linear accelerator); each additional cranial lesion, complex (List separately in addition to code for primary procedure)
		procedure	UMLS:CPT:61800	Application of stereotactic headframe for stereotactic radiosurgery (List separately in addition to code for primary procedure)

procedure	UMLS:CPT:77371	Radiation treatment delivery, stereotactic radiosurgery (SRS), complete course of treatment of cranial lesion(s) consisting of 1 session; multi-source Cobalt 60 based
procedure	UMLS:CPT:77372	Radiation treatment delivery, stereotactic radiosurgery (SRS), complete course of treatment of cranial lesion(s) consisting of 1 session; linear accelerator based
procedure	UMLS:CPT:77432	Stereotactic radiation treatment management of cranial lesion(s) (complete course of treatment consisting of 1 session)
procedure	UMLS:HCPCS:G0339	Image-guided robotic linear accelerator-based stereotactic radiosurgery, complete course of therapy in one session or first session of fractionated treatment
procedure	UMLS:HCPCS:G0340	Image-guided robotic linear accelerator-based stereotactic radiosurgery, delivery including collimator changes and custom plugging, fractionated treatment, all lesions, per session, second through fifth sessions, maximum five sessions per course of treatment
procedure	UMLS:CPT:77401	Radiation treatment delivery, superficial and/or ortho voltage, per day
procedure	UMLS:CPT:77402	Radiation treatment delivery, >=1 MeV; simple
procedure	UMLS:CPT:77407	Radiation treatment delivery, >=1 MeV; intermediate
procedure	UMLS:CPT:77412	Radiation treatment delivery, >=1 MeV; complex
procedure	UMLS:HCPCS:G6004	Radiation treatment delivery, single treatment area,single port or parallel opposed ports, simple blocks or no blocks: 6-10 mev
procedure	UMLS:HCPCS:G6005	Radiation treatment delivery, single treatment area,single port or parallel opposed ports, simple blocks or no blocks: 11-19 mev
procedure	UMLS:HCPCS:G6006	Radiation treatment delivery, single treatment area,single port or parallel opposed ports, simple blocks or no blocks: 20 mev or greater
procedure	UMLS:HCPCS:G6007	Radiation treatment delivery, 2 separate treatment areas, 3 or more ports on a single treatment area, use of multiple blocks: up to 5 mev
procedure	UMLS:HCPCS:G6008	Radiation treatment delivery, 2 separate treatment areas, 3 or more ports on a single treatment area, use of multiple blocks: 6-10 mev
procedure	UMLS:HCPCS:G6009	Radiation treatment delivery, 2 separate treatment areas, 3 or more

				ports on a single treatment area, use of multiple blocks: 11-19 mev
		procedure	UMLS:HCPCS:G6010	Radiation treatment delivery, 2 separate treatment areas, 3 or more ports on a single treatment area, use of multiple blocks: 20 mev or greater
		procedure	UMLS:HCPCS:G6011	Radiation treatment delivery,3 or more separate treatment areas, custom blocking, tangential ports, wedges, rotational beam, compensators, electron beam; up to 5 mev
		procedure	UMLS:HCPCS:G6012	Radiation treatment delivery,3 or more separate treatment areas, custom blocking, tangential ports, wedges, rotational beam, compensators, electron beam; 6-10 mev
		procedure	UMLS:HCPCS:G6013	Radiation treatment delivery,3 or more separate treatment areas, custom blocking, tangential ports, wedges, rotational beam, compensators, electron beam; 11-19 mev
		procedure	UMLS:HCPCS:G6014	Radiation treatment delivery,3 or more separate treatment areas, custom blocking, tangential ports, wedges, rotational beam, compensators, electron beam; 20 mev or greater
		procedure	UMLS:HCPCS:G6016	Compensator-based beam modulation treatment delivery of inverse planned treatment using 3 or more high resolution (milled or cast) compensator, convergent beam modulated fields, per treatment session
		procedure	UMLS:CPT:77373	Stereotactic body radiation therapy, treatment delivery, per fraction to 1 or more lesions, including image guidance, entire course not to exceed 5 fractions
**Group 5**				
**Group 5A ICI**				
must have	any of	procedure	UMLS:HCPCS:J9271	Injection, pembrolizumab, 1 mg
		procedure	UMLS:HCPCS:C9027	Injection, pembrolizumab, 1 mg (deprecated 2015)
		procedure	UMLS:HCPCS:J9299	Injection, nivolumab, 1 mg
		procedure	UMLS:HCPCS:J9298	Injection, nivolumab and relatlimab-rmbw, 3 mg/1 mg
		procedure	UMLS:HCPCS:C9453	Injection, nivolumab, 1 mg (deprecated 2015)
		procedure	UMLS:HCPCS:J9228	Injection, ipilimumab, 1 mg
		procedure	UMLS:HCPCS:J9022	Injection, atezolizumab, 10 mg
		procedure	UMLS:HCPCS:C9483	Injection, atezolizumab, 10 mg (deprecated 2017)
		procedure	UMLS:HCPCS:J9023	Injection, avelumab, 10 mg
		procedure	UMLS:HCPCS:C9491	Injection, avelumab, 10 mg (deprecated 2017)
		procedure	UMLS:HCPCS:J9173	Injection, durvalumab, 10 mg
		procedure	UMLS:HCPCS:J9119	Injection, cemiplimab-rwlc, 1 mg
		procedure	UMLS:HCPCS:C9492	Injection, durvalumab, 10 mg (deprecated 2018)

		procedure	UMLS:HCPCS:C9044	Injection, cemiplimab-rwlc, 1 mg (deprecated 2019)
date constraint		The terms in this group occurred at any time		
event relationship		Any instance of CR occurred within 6 months and 31 days before any instance of ICI		
**Group 5B CR**				
must have	any of	procedure	UMLS:CPT:61796	Stereotactic radiosurgery (particle beam, gamma ray, or linear accelerator); 1 simple cranial lesion
		procedure	UMLS:CPT:61798	Stereotactic radiosurgery (particle beam, gamma ray, or linear accelerator); 1 complex cranial lesion
		procedure	UMLS:CPT:61799	Stereotactic radiosurgery (particle beam, gamma ray, or linear accelerator); each additional cranial lesion, complex (List separately in addition to code for primary procedure)
		procedure	UMLS:CPT:61800	Application of stereotactic headframe for stereotactic radiosurgery (List separately in addition to code for primary procedure)
		procedure	UMLS:CPT:77371	Radiation treatment delivery, stereotactic radiosurgery (SRS), complete course of treatment of cranial lesion(s) consisting of 1 session; multi-source Cobalt 60 based
		procedure	UMLS:CPT:77372	Radiation treatment delivery, stereotactic radiosurgery (SRS), complete course of treatment of cranial lesion(s) consisting of 1 session; linear accelerator based
		procedure	UMLS:CPT:77432	Stereotactic radiation treatment management of cranial lesion(s) (complete course of treatment consisting of 1 session)
		procedure	UMLS:HCPCS:G0339	Image-guided robotic linear accelerator-based stereotactic radiosurgery, complete course of therapy in one session or first session of fractionated treatment
		procedure	UMLS:HCPCS:G0340	Image-guided robotic linear accelerator-based stereotactic radiosurgery, delivery including collimator changes and custom plugging, fractionated treatment, all lesions, per session, second through fifth sessions, maximum five sessions per course of treatment
		procedure	UMLS:CPT:77401	Radiation treatment delivery, superficial and/or ortho voltage, per day
		procedure	UMLS:CPT:77402	Radiation treatment delivery, >=1 MeV; simple
		procedure	UMLS:CPT:77407	Radiation treatment delivery, >=1 MeV; intermediate
		procedure	UMLS:CPT:77412	Radiation treatment delivery, >=1 MeV; complex
		procedure	UMLS:HCPCS:G6004	Radiation treatment delivery, single treatment area,single port or parallel

		opposed ports, simple blocks or no blocks: 6-10 mev
procedure	UMLS:HCPCS:G6005	Radiation treatment delivery, single treatment area,single port or parallel opposed ports, simple blocks or no blocks: 11-19 mev
procedure	UMLS:HCPCS:G6006	Radiation treatment delivery, single treatment area,single port or parallel opposed ports, simple blocks or no blocks: 20 mev or greater
procedure	UMLS:HCPCS:G6007	Radiation treatment delivery, 2 separate treatment areas, 3 or more ports on a single treatment area, use of multiple blocks: up to 5 mev
procedure	UMLS:HCPCS:G6008	Radiation treatment delivery, 2 separate treatment areas, 3 or more ports on a single treatment area, use of multiple blocks: 6-10 mev
procedure	UMLS:HCPCS:G6009	Radiation treatment delivery, 2 separate treatment areas, 3 or more ports on a single treatment area, use of multiple blocks: 11-19 mev
procedure	UMLS:HCPCS:G6010	Radiation treatment delivery, 2 separate treatment areas, 3 or more ports on a single treatment area, use of multiple blocks: 20 mev or greater
procedure	UMLS:HCPCS:G6011	Radiation treatment delivery,3 or more separate treatment areas, custom blocking, tangential ports, wedges, rotational beam, compensators, electron beam; up to 5 mev
procedure	UMLS:HCPCS:G6012	Radiation treatment delivery,3 or more separate treatment areas, custom blocking, tangential ports, wedges, rotational beam, compensators, electron beam; 6-10 mev
procedure	UMLS:HCPCS:G6013	Radiation treatment delivery,3 or more separate treatment areas, custom blocking, tangential ports, wedges, rotational beam, compensators, electron beam; 11-19 mev
procedure	UMLS:HCPCS:G6014	Radiation treatment delivery,3 or more separate treatment areas, custom blocking, tangential ports, wedges, rotational beam, compensators, electron beam; 20 mev or greater
procedure	UMLS:HCPCS:G6016	Compensator-based beam modulation treatment delivery of inverse planned treatment using 3 or more high resolution (milled or cast) compensator, convergent beam modulated fields, per treatment session
procedure	UMLS:CPT:77373	Stereotactic body radiation therapy, treatment delivery, per fraction to 1 or more lesions, including image guidance, entire course not to exceed 5 fractions

Analysis Setup

This section contains the Index Event and Time Window definitions and a list of selected outcomes and the analyses.

Index Event & Time Window Definitions

The index event defines the point in time when each patient in the cohort enters the analysis. To define an index event for the cohort, one or more criteria for the cohort must be selected. The index date for each patient within a cohort is the day on which the patient first met the selected criteria for the cohort (listed in the table below).

As the index event defines the earliest time point after which outcomes are analyzed, the time window defines the duration during which outcomes are analyzed. The time window can start on the same day as the index event or at any specified time interval after the index event. The time window can end any time after the start date. Outcomes are defined as diagnoses, medications, procedures, or laboratory values that happened in the time window starting after the first occurrence of the index event.

Time Window Used in this Analysis

This analysis included outcomes that occurred in the time window that started 1 day after the first occurrence of the index event. Since no end date was specified all outcomes after the first occurrence of the index event were included.

The index event only includes events that occurred up to 20 years ago. Patients whose index event occurred 20 years or more ago are excluded. In this analysis, 0 patients in Cohort 1 and 0 patients in Cohort 2 were excluded because they met the index event more than 20 years ago.

Index Events Used in this Analysis

Index events for the Compare Outcomes analysis were derived from the cohort definitions. Index events were defined separately for each cohort and were based on the criteria used in the original cohort definition. Please see Appendix B for the text representation of the index event definition.

The index event for Cohort 1 (query name: ICI-CR 0-15 days) was defined as the following:

**Group 1**					
	**Group 1A ICI**				
	must have	any of	procedure	UMLS:HCPCS:J9271	Injection, pembrolizumab, 1 mg
			procedure	UMLS:HCPCS:C9027	Injection, pembrolizumab, 1 mg (deprecated 2015)
			procedure	UMLS:HCPCS:J9299	Injection, nivolumab, 1 mg
			procedure	UMLS:HCPCS:J9298	Injection, nivolumab and relatlimab-rmbw, 3 mg/1 mg
			procedure	UMLS:HCPCS:C9453	Injection, nivolumab, 1 mg (deprecated 2015)
			procedure	UMLS:HCPCS:J9228	Injection, ipilimumab, 1 mg
			procedure	UMLS:HCPCS:J9022	Injection, atezolizumab, 10 mg
			procedure	UMLS:HCPCS:C9483	Injection, atezolizumab, 10 mg (deprecated 2017)
			procedure	UMLS:HCPCS:J9023	Injection, avelumab, 10 mg
			procedure	UMLS:HCPCS:C9491	Injection, avelumab, 10 mg (deprecated 2017)
			procedure	UMLS:HCPCS:J9173	Injection, durvalumab, 10 mg
			procedure	UMLS:HCPCS:J9119	Injection, cemiplimab-rwlc, 1 mg

		procedure	UMLS:HCPCS:C9492	Injection, durvalumab, 10 mg (deprecated 2018)
		procedure	UMLS:HCPCS:C9044	Injection, cemiplimab-rwlc, 1 mg (deprecated 2019)
date constraint		The terms in this group occurred at any time		
event relationship		Any instance of CR occurred within 15 days before or up to 15 days after any instance of ICI		
**Group 1B CR**				
must have	any of	procedure	UMLS:CPT:61796	Stereotactic radiosurgery (particle beam, gamma ray, or linear accelerator); 1 simple cranial lesion
		procedure	UMLS:CPT:61798	Stereotactic radiosurgery (particle beam, gamma ray, or linear accelerator); 1 complex cranial lesion
		procedure	UMLS:CPT:61799	Stereotactic radiosurgery (particle beam, gamma ray, or linear accelerator); each additional cranial lesion, complex (List separately in addition to code for primary procedure)
		procedure	UMLS:CPT:61800	Application of stereotactic headframe for stereotactic radiosurgery (List separately in addition to code for primary procedure)
		procedure	UMLS:CPT:77371	Radiation treatment delivery, stereotactic radiosurgery (SRS), complete course of treatment of cranial lesion(s) consisting of 1 session; multi-source Cobalt 60 based
		procedure	UMLS:CPT:77372	Radiation treatment delivery, stereotactic radiosurgery (SRS), complete course of treatment of cranial lesion(s) consisting of 1 session; linear accelerator based
		procedure	UMLS:CPT:77432	Stereotactic radiation treatment management of cranial lesion(s) (complete course of treatment consisting of 1 session)
		procedure	UMLS:HCPCS:G0339	Image-guided robotic linear accelerator-based stereotactic radiosurgery, complete course of therapy in one session or first session of fractionated treatment
		procedure	UMLS:HCPCS:G0340	Image-guided robotic linear accelerator-based stereotactic radiosurgery, delivery including collimator changes and custom plugging, fractionated treatment, all lesions, per session, second through fifth sessions, maximum five sessions per course of treatment
		procedure	UMLS:CPT:77401	Radiation treatment delivery, superficial and/or ortho voltage, per day
		procedure	UMLS:CPT:77402	Radiation treatment delivery, >=1 MeV; simple
		procedure	UMLS:CPT:77407	Radiation treatment delivery, >=1 MeV; intermediate
		procedure	UMLS:CPT:77412	Radiation treatment delivery, >=1 MeV; complex

procedure	UMLS:HCPCS:G6004	Radiation treatment delivery, single treatment area,single port or parallel opposed ports, simple blocks or no blocks: 6-10 mev
procedure	UMLS:HCPCS:G6005	Radiation treatment delivery, single treatment area,single port or parallel opposed ports, simple blocks or no blocks: 11-19 mev
procedure	UMLS:HCPCS:G6006	Radiation treatment delivery, single treatment area,single port or parallel opposed ports, simple blocks or no blocks: 20 mev or greater
procedure	UMLS:HCPCS:G6007	Radiation treatment delivery, 2 separate treatment areas, 3 or more ports on a single treatment area, use of multiple blocks: up to 5 mev
procedure	UMLS:HCPCS:G6008	Radiation treatment delivery, 2 separate treatment areas, 3 or more ports on a single treatment area, use of multiple blocks: 6-10 mev
procedure	UMLS:HCPCS:G6009	Radiation treatment delivery, 2 separate treatment areas, 3 or more ports on a single treatment area, use of multiple blocks: 11-19 mev
procedure	UMLS:HCPCS:G6010	Radiation treatment delivery, 2 separate treatment areas, 3 or more ports on a single treatment area, use of multiple blocks: 20 mev or greater
procedure	UMLS:HCPCS:G6011	Radiation treatment delivery,3 or more separate treatment areas, custom blocking, tangential ports, wedges, rotational beam, compensators, electron beam; up to 5 mev
procedure	UMLS:HCPCS:G6012	Radiation treatment delivery,3 or more separate treatment areas, custom blocking, tangential ports, wedges, rotational beam, compensators, electron beam; 6-10 mev
procedure	UMLS:HCPCS:G6013	Radiation treatment delivery,3 or more separate treatment areas, custom blocking, tangential ports, wedges, rotational beam, compensators, electron beam; 11-19 mev
procedure	UMLS:HCPCS:G6014	Radiation treatment delivery,3 or more separate treatment areas, custom blocking, tangential ports, wedges, rotational beam, compensators, electron beam; 20 mev or greater
procedure	UMLS:HCPCS:G6016	Compensator-based beam modulation treatment delivery of inverse planned treatment using 3 or more high resolution (milled or cast) compensator, convergent beam modulated fields, per treatment session
procedure	UMLS:CPT:77373	Stereotactic body radiation therapy, treatment delivery, per fraction to 1 or more lesions, including image guidance, entire course not to exceed 5 fractions

The index event for Cohort 2 (query name: ICI-CR 31+days new) was defined as the following:

**Group 1 or Group 2 must be present**				
**Group 1**				
**Group 1A ICI**				
must have	any of	procedure	UMLS:HCPCS:J9271	Injection, pembrolizumab, 1 mg
		procedure	UMLS:HCPCS:C9027	Injection, pembrolizumab, 1 mg (deprecated 2015)
		procedure	UMLS:HCPCS:J9299	Injection, nivolumab, 1 mg
		procedure	UMLS:HCPCS:J9298	Injection, nivolumab and relatlimab-rmbw, 3 mg/1 mg
		procedure	UMLS:HCPCS:C9453	Injection, nivolumab, 1 mg (deprecated 2015)
		procedure	UMLS:HCPCS:J9228	Injection, ipilimumab, 1 mg
		procedure	UMLS:HCPCS:J9022	Injection, atezolizumab, 10 mg
		procedure	UMLS:HCPCS:C9483	Injection, atezolizumab, 10 mg (deprecated 2017)
		procedure	UMLS:HCPCS:J9023	Injection, avelumab, 10 mg
		procedure	UMLS:HCPCS:C9491	Injection, avelumab, 10 mg (deprecated 2017)
		procedure	UMLS:HCPCS:J9173	Injection, durvalumab, 10 mg
		procedure	UMLS:HCPCS:J9119	Injection, cemiplimab-rwlc, 1 mg
		procedure	UMLS:HCPCS:C9492	Injection, durvalumab, 10 mg (deprecated 2018)
		procedure	UMLS:HCPCS:C9044	Injection, cemiplimab-rwlc, 1 mg (deprecated 2019)
date constraint		The terms in this group occurred at any time		
event relationship		Any instance of CR occurred within 31 days and 6 months after any instance of ICI		
**Group 1B CR**				
must have	any of	procedure	UMLS:CPT:61796	Stereotactic radiosurgery (particle beam, gamma ray, or linear accelerator); 1 simple cranial lesion
		procedure	UMLS:CPT:61798	Stereotactic radiosurgery (particle beam, gamma ray, or linear accelerator); 1 complex cranial lesion
		procedure	UMLS:CPT:61799	Stereotactic radiosurgery (particle beam, gamma ray, or linear accelerator); each additional cranial lesion, complex (List separately in addition to code for primary procedure)
		procedure	UMLS:CPT:61800	Application of stereotactic headframe for stereotactic radiosurgery (List separately in addition to code for primary procedure)
		procedure	UMLS:CPT:77371	Radiation treatment delivery, stereotactic radiosurgery (SRS), complete course of treatment of cranial lesion(s) consisting of 1 session; multi-source Cobalt 60 based
		procedure	UMLS:CPT:77372	Radiation treatment delivery, stereotactic radiosurgery (SRS), complete course of treatment of cranial lesion(s) consisting of 1 session; linear accelerator based
		procedure	UMLS:CPT:77432	Stereotactic radiation treatment management of cranial lesion(s)

		(complete course of treatment consisting of 1 session)
procedure	UMLS:HCPCS:G0339	Image-guided robotic linear accelerator-based stereotactic radiosurgery, complete course of therapy in one session or first session of fractionated treatment
procedure	UMLS:HCPCS:G0340	Image-guided robotic linear accelerator-based stereotactic radiosurgery, delivery including collimator changes and custom plugging, fractionated treatment, all lesions, per session, second through fifth sessions, maximum five sessions per course of treatment
procedure	UMLS:CPT:77401	Radiation treatment delivery, superficial and/or ortho voltage, per day
procedure	UMLS:CPT:77402	Radiation treatment delivery, >=1 MeV; simple
procedure	UMLS:CPT:77407	Radiation treatment delivery, >=1 MeV; intermediate
procedure	UMLS:CPT:77412	Radiation treatment delivery, >=1 MeV; complex
procedure	UMLS:HCPCS:G6004	Radiation treatment delivery, single treatment area,single port or parallel opposed ports, simple blocks or no blocks: 6-10 mev
procedure	UMLS:HCPCS:G6005	Radiation treatment delivery, single treatment area,single port or parallel opposed ports, simple blocks or no blocks: 11-19 mev
procedure	UMLS:HCPCS:G6006	Radiation treatment delivery, single treatment area,single port or parallel opposed ports, simple blocks or no blocks: 20 mev or greater
procedure	UMLS:HCPCS:G6007	Radiation treatment delivery, 2 separate treatment areas, 3 or more ports on a single treatment area, use of multiple blocks: up to 5 mev
procedure	UMLS:HCPCS:G6008	Radiation treatment delivery, 2 separate treatment areas, 3 or more ports on a single treatment area, use of multiple blocks: 6-10 mev
procedure	UMLS:HCPCS:G6009	Radiation treatment delivery, 2 separate treatment areas, 3 or more ports on a single treatment area, use of multiple blocks: 11-19 mev
procedure	UMLS:HCPCS:G6010	Radiation treatment delivery, 2 separate treatment areas, 3 or more ports on a single treatment area, use of multiple blocks: 20 mev or greater
procedure	UMLS:HCPCS:G6011	Radiation treatment delivery,3 or more separate treatment areas, custom blocking, tangential ports, wedges, rotational beam, compensators, electron beam; up to 5 mev
procedure	UMLS:HCPCS:G6012	Radiation treatment delivery,3 or more separate treatment areas, custom

				blocking, tangential ports, wedges, rotational beam, compensators, electron beam; 6-10 mev
		procedure	UMLS:HCPCS:G6013	Radiation treatment delivery,3 or more separate treatment areas, custom blocking, tangential ports, wedges, rotational beam, compensators, electron beam; 11-19 mev
		procedure	UMLS:HCPCS:G6014	Radiation treatment delivery,3 or more separate treatment areas, custom blocking, tangential ports, wedges, rotational beam, compensators, electron beam; 20 mev or greater
		procedure	UMLS:HCPCS:G6016	Compensator-based beam modulation treatment delivery of inverse planned treatment using 3 or more high resolution (milled or cast) compensator, convergent beam modulated fields, per treatment session
		procedure	UMLS:CPT:77373	Stereotactic body radiation therapy, treatment delivery, per fraction to 1 or more lesions, including image guidance, entire course not to exceed 5 fractions
**Group 2**				
**Group 2A ICI**				
must have	any of	procedure	UMLS:HCPCS:J9271	Injection, pembrolizumab, 1 mg
		procedure	UMLS:HCPCS:C9027	Injection, pembrolizumab, 1 mg (deprecated 2015)
		procedure	UMLS:HCPCS:J9299	Injection, nivolumab, 1 mg
		procedure	UMLS:HCPCS:J9298	Injection, nivolumab and relatlimab-rmbw, 3 mg/1 mg
		procedure	UMLS:HCPCS:C9453	Injection, nivolumab, 1 mg (deprecated 2015)
		procedure	UMLS:HCPCS:J9228	Injection, ipilimumab, 1 mg
		procedure	UMLS:HCPCS:J9022	Injection, atezolizumab, 10 mg
		procedure	UMLS:HCPCS:C9483	Injection, atezolizumab, 10 mg (deprecated 2017)
		procedure	UMLS:HCPCS:J9023	Injection, avelumab, 10 mg
		procedure	UMLS:HCPCS:C9491	Injection, avelumab, 10 mg (deprecated 2017)
		procedure	UMLS:HCPCS:J9173	Injection, durvalumab, 10 mg
		procedure	UMLS:HCPCS:J9119	Injection, cemiplimab-rwlc, 1 mg
		procedure	UMLS:HCPCS:C9492	Injection, durvalumab, 10 mg (deprecated 2018)
		procedure	UMLS:HCPCS:C9044	Injection, cemiplimab-rwlc, 1 mg (deprecated 2019)
date constraint		The terms in this group occurred at any time		
event relationship		Any instance of CR occurred within 6 months and 31 days before any instance of ICI		
**Group 2B CR**				
must have	any of	procedure	UMLS:CPT:61796	Stereotactic radiosurgery (particle beam, gamma ray, or linear accelerator); 1 simple cranial lesion
		procedure	UMLS:CPT:61798	Stereotactic radiosurgery (particle beam, gamma ray, or linear accelerator); 1 complex cranial lesion
		procedure	UMLS:CPT:61799	Stereotactic radiosurgery (particle beam, gamma ray, or linear accelerator); each additional cranial

		lesion, complex (List separately in addition to code for primary procedure)
procedure	UMLS:CPT:61800	Application of stereotactic headframe for stereotactic radiosurgery (List separately in addition to code for primary procedure)
procedure	UMLS:CPT:77371	Radiation treatment delivery, stereotactic radiosurgery (SRS), complete course of treatment of cranial lesion(s) consisting of 1 session; multi-source Cobalt 60 based
procedure	UMLS:CPT:77372	Radiation treatment delivery, stereotactic radiosurgery (SRS), complete course of treatment of cranial lesion(s) consisting of 1 session; linear accelerator based
procedure	UMLS:CPT:77432	Stereotactic radiation treatment management of cranial lesion(s) (complete course of treatment consisting of 1 session)
procedure	UMLS:HCPCS:G0339	Image-guided robotic linear accelerator-based stereotactic radiosurgery, complete course of therapy in one session or first session of fractionated treatment
procedure	UMLS:HCPCS:G0340	Image-guided robotic linear accelerator-based stereotactic radiosurgery, delivery including collimator changes and custom plugging, fractionated treatment, all lesions, per session, second through fifth sessions, maximum five sessions per course of treatment
procedure	UMLS:CPT:77401	Radiation treatment delivery, superficial and/or ortho voltage, per day
procedure	UMLS:CPT:77402	Radiation treatment delivery, >=1 MeV; simple
procedure	UMLS:CPT:77407	Radiation treatment delivery, >=1 MeV; intermediate
procedure	UMLS:CPT:77412	Radiation treatment delivery, >=1 MeV; complex
procedure	UMLS:HCPCS:G6004	Radiation treatment delivery, single treatment area,single port or parallel opposed ports, simple blocks or no blocks: 6-10 mev
procedure	UMLS:HCPCS:G6005	Radiation treatment delivery, single treatment area,single port or parallel opposed ports, simple blocks or no blocks: 11-19 mev
procedure	UMLS:HCPCS:G6006	Radiation treatment delivery, single treatment area,single port or parallel opposed ports, simple blocks or no blocks: 20 mev or greater
procedure	UMLS:HCPCS:G6007	Radiation treatment delivery, 2 separate treatment areas, 3 or more ports on a single treatment area, use of multiple blocks: up to 5 mev

procedure	UMLS:HCPCS:G6008	Radiation treatment delivery, 2 separate treatment areas, 3 or more ports on a single treatment area, use of multiple blocks: 6-10 mev
procedure	UMLS:HCPCS:G6009	Radiation treatment delivery, 2 separate treatment areas, 3 or more ports on a single treatment area, use of multiple blocks: 11-19 mev
procedure	UMLS:HCPCS:G6010	Radiation treatment delivery, 2 separate treatment areas, 3 or more ports on a single treatment area, use of multiple blocks: 20 mev or greater
procedure	UMLS:HCPCS:G6011	Radiation treatment delivery,3 or more separate treatment areas, custom blocking, tangential ports, wedges, rotational beam, compensators, electron beam; up to 5 mev
procedure	UMLS:HCPCS:G6012	Radiation treatment delivery,3 or more separate treatment areas, custom blocking, tangential ports, wedges, rotational beam, compensators, electron beam; 6-10 mev
procedure	UMLS:HCPCS:G6013	Radiation treatment delivery,3 or more separate treatment areas, custom blocking, tangential ports, wedges, rotational beam, compensators, electron beam; 11-19 mev
procedure	UMLS:HCPCS:G6014	Radiation treatment delivery,3 or more separate treatment areas, custom blocking, tangential ports, wedges, rotational beam, compensators, electron beam; 20 mev or greater
procedure	UMLS:HCPCS:G6016	Compensator-based beam modulation treatment delivery of inverse planned treatment using 3 or more high resolution (milled or cast) compensator, convergent beam modulated fields, per treatment session
procedure	UMLS:CPT:77373	Stereotactic body radiation therapy, treatment delivery, per fraction to 1 or more lesions, including image guidance, entire course not to exceed 5 fractions

Analyses Specifications

The Compare Outcomes Analytic supports four types of analyses: Measure of Association, Survival, Number of Instances, and Lab result distribution. The first three analyses support the “exclude patients with outcomes prior to the window” setting. This option can exclude patients from the analysis if they are not at risk for an outcome (e.g., if the outcome is a chronic disease). When "exclude patients with the outcome prior to the time window" is not checked, all patients in the cohort are included in the analysis, regardless of whether they had the outcome prior to the time window. When "exclude patients with the outcome prior to the time window" is checked, patients are excluded from the analysis if their record includes the outcome prior to the beginning of the time window. This selection will exclude all patients with the outcome prior to the index event. If the start of the time window for the analysis falls some days after the index event, patients will also be excluded if they have the outcome between the index event and the start of the time window.

Measure of Association Analysis

The Measure of Association Analysis calculates and compares the fraction of patients with the selected outcome. The output summary includes: Patients in each Cohort (count of patients meeting query criteria); Patients with Outcome in each Cohort (of the patients in the cohort, count of patients that had the outcome in the time window); and Risk (the fraction of patients in the cohort that have the outcome in the time window, i.e. Patients with Outcome / Patients in Cohort). In addition, Risk Difference (the difference in the risks in Cohort 1 and Cohort 2), Risk Ratio (the ratio of the risks in Cohort 1 and Cohort 2), and Odds Ratio (the ratio of the odds in Cohort 1 and Cohort 2). The bar chart shows the risk of the outcome for the both cohorts.

Survival Analysis

The Kaplan-Meier Analysis estimates probability of the outcome at a respective time interval (daily time interval is used in this analysis). In order to account for the patients who exited the cohort during the analysis period, and therefore should not be included in the analysis, censoring is applied. In this analysis, patients are removed from the analysis (censored) after the last fact in their record.

The output summary includes: Patients in each Cohort (count of patients meeting query criteria); Patients with Outcome (of the patients in the cohort, count of patients that had the outcome in the time window); Median Survival (the number of days when the survival drops below 50%; the "-" indicates that survival does not drop below 50% during the time window); and Survival Probability at End of Time Window (the % survival at the end of the time window). In addition, Log-Rank test, Hazard Ratio and test for Proportionality.

Number of Instances Analysis

The Number of Instances Analysis calculates how many times the outcome occurred in the time window. This analysis includes two additional settings: include patients with zero instances; the definition of an instance.

Selecting to exclude patients with zero instances will remove these patients from the calculations for mean number of instances, standard deviation, or median. The histogram showing the distribution of patients by number of instances will not contain a bar for zero. Alternatively, by selecting to include patients with zero instances, the mean, standard deviation, and median for number of instances will reflect these patients. The histogram will contain a bar for zero patients.

The definition of an instance affects how counts are analyzed. By selecting Date, each calendar date on which any of the terms selected in the outcome are recorded will represent one instance. For example, if the outcome is "Med A or Med B," and a patient has "Med A" on January 3, then both medications on January 4, then "Med B" on January 6, then that patient is considered to have three instances– January 3, January 4, and January 6. Note that if an outcome occurs across several dates (e.g. Visit: inpatient encounter), then only the start date is tracked for the purpose of counting instances. A patient who begins at stay on January 1, ends that stay on January 3, begins another stay on January 10, and ends that stay on January 15, is considered to have two instances of the outcome.

Selecting Visit as an Instance will count any visit that includes the outcome as one instance, regardless of how many times it occurred. For instance, consider a patient administered an analgesic on each of the three days that make up an inpatient stay following some index event. If analgesic is an outcome, these three administrations will represent only one instance, because all three are associated with the same visit.

The output summary includes: Patients in Cohort (count of patients meeting query criteria); Patients with Outcome (of the patients in the cohort, count of patients that had the outcome in the time window); Mean

(mean of the counts); Standard Deviation (standard deviation of the counts); Median (median of the counts); and Median (1+ instances) when patients with zero instances included in the analysis. In addition, T-Test statistics testing for the difference between the cohorts is included.

Laboratory Results Analysis

Lab Results can be included in the analysis only for the outcomes that are labs. Only the most recent lab values in the time window are included. For the lab results that are numeric, the outcome summary includes: Patients in Cohort (count of patients meeting query criteria); Patients with Outcome (of the patients in the cohort, count of patients that had the outcome in the time window); Mean (mean of the counts); and Standard Deviation (the standard deviation for lab values across patients in the cohort). In addition, T-Test statistics testing for the difference between the cohorts is included.

For the non-numeric lab results, three values are reported: counts of Negative; Positives; and Unknowns. The counts are represented in the bar chart as percentages of the total counts.

Outcome Definitions

Table below outlines the definitions for each outcome and the analysis specifications. For outcome definitions consisting of more than one term, at least one term must match. Please see Appendix C for the text representation of the outcome definitions.

**Unnamed Outcome**		
**Outcome definition**		
Diagnosis	UMLS:ICD10CM:R99	Ill-defined and unknown cause of mortality
Diagnosis	UMLS:ICD10CM:R99-R99	Ill-defined and unknown cause of mortality (R99)
Diagnosis	UMLS:ICD10CM:R69	Illness, unspecified
Demographics	Deceased	Deceased
**Settings for the performed analyses**		
Kaplan - Meier survival analysis		excluding patients with outcome prior to the time window
Number of instances analysis		including patients with outcome prior to the time window<br>including patients with zero outcomes<br>counts are grouped by date

Propensity Score Matching

Propensity score matching was not used in this analysis.

Results

Results are summarized in the tables below.

**Follow-up Time**				
Cohort	Mean Follow-up (Days)	Standard Deviation	Median Follow-up (Days)	Interquartile Range
ICI-CR 0-15 days	592.314	630.491	348.500	683.500
ICI-CR 31+days new	611.406	658.686	348	694

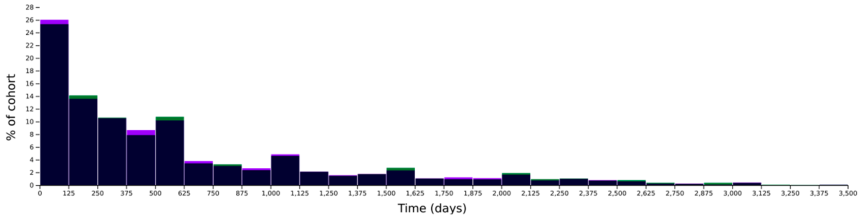

1 Overall Survival

Kaplan - Meier survival analysis excluding patients with outcome prior to the time window

Cohort	Patients in cohort	Patients with outcome	Median survival (days)	Survival probability at end of time window
1 ICI-CR 0-15 days	2,397	1,565	362	11.85%
2 ICI-CR >31 days new	3,603	2,336	366	14.16%

	χ^2	df	p
**Log-Rank Test**	0.587	1	0.444

	Hazard Ratio	95% CI	χ^2	df	p
**Hazard Ratio and Proportionality**	1.025	(0.962, 1.093)	0.420	1	0.517

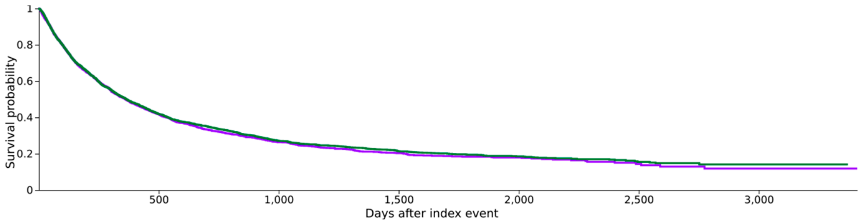

167 patients in Cohort 1 and 259 patients in Cohort 2 were excluded from results because they had the outcome prior to the time window.

Number of instances

Cohort	Patients in cohort	Patients with outcome	Mean	Standard Deviation	Median	Median (1+ instances)
1 ICI-CR 0-15 days	2,564	1,687	0.789	0.700	1	1
2 ICI-CR 31+days new	3,862	2,531	0.779	0.667	1	1

	t	df	p
**Test Statistics**	0.570	6424	0.569

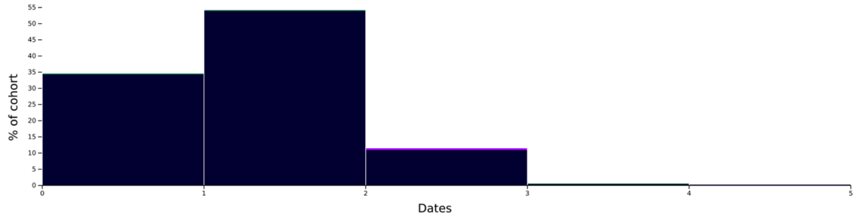

3 data points for Cohort 1 and 2 data points for Cohort 2 were omitted for display purposes.
Patients with zero instances included in calculations. Percentages in chart based on patients in cohort.

Appendix A – Text Representation of the Cohorts Definition

This section lists all terms used in the definitions of the two cohorts.

Query Criteria for Cohort 1 (query name: ICI-CR 0-15 days)

Patients must have:
  Age (Age) (between 65 and 120 years (most recent occurrence)).

Patients cannot have:
  any of the following:
    ceritinib (NLM:RXNORM:1535457); or
    afatinib (NLM:RXNORM:1430438); or
    alectinib (NLM:RXNORM:1727455); or
    crizotinib (NLM:RXNORM:1148495); or
    brigatinib (NLM:RXNORM:1921217); or
    erlotinib (NLM:RXNORM:337525); or
    dabrafenib (NLM:RXNORM:1424911); or
    gefitinib (NLM:RXNORM:328134); or
    osimertinib (NLM:RXNORM:1721560); or
    trametinib (NLM:RXNORM:1425099).

All the following must be satisfied:

  NSCLC DX: The terms in this group occurred at any time
    Patients must have:
      any of the following:
        Malignant neoplasm of bronchus and lung (UMLS:ICD10CM:C34); or
        Bronchus and lung (UMLS:ICDO3:C34); or
        Malignant neoplasm of bronchus and lung (UMLS:ICD10CM:C34); or
        Stage 1 (TNX:STAGE:S1); or
        Stage 2 (TNX:STAGE:S2); or
        Stage 3 (TNX:STAGE:S3); or
        Non-small cell carcinoma (TNX:TNX:NSCC); or
        Malignant neoplasm of bronchus and lung (UMLS:ICD10CM:C34); or
        CD274 (GENE:17635); or
        Cells.programmed cell death ligand 1/100 viable tumor cells in Tissue by Immune stain (UMLS:LNC:83053-9).
    Patients cannot have:
      any of the following:
        Small cell carcinoma (TNX:TNX:SCC); or
        Small cell carcinoma, NOS (UMLS:ICDO3:8041/3); or
        Combined small cell carcinoma (UMLS:ICDO3:8045/3); or
        Small cell carcinoma, fusiform cell (UMLS:ICDO3:8043/3); or
        Small cell carcinoma, intermediate cell (UMLS:ICDO3:8044/3); or
        EGFR (GENE:3236); or
        ALK (GENE:427).
  BM DX: Any instance of BM DX occurred within 1 day before or up to 1 day after any instance of NSCLC DX
    Patients must have:
      any of the following:

Secondary malignant neoplasm of brain (UMLS:ICD10CM:C79.31); or
Stage 4 (TNX:STAGE:S4); or
M1 (TNX:STAGE:TNM_M1).

ICI: The terms in this group occurred at any time
Patients must have:
any of the following:
Injection, pembrolizumab, 1 mg (UMLS:HCPCS:J9271); or
Injection, pembrolizumab, 1 mg (deprecated 2015) (UMLS:HCPCS:C9027); or
Injection, nivolumab, 1 mg (UMLS:HCPCS:J9299); or
Injection, nivolumab and relatlimab-rmbw, 3 mg/1 mg (UMLS:HCPCS:J9298); or
Injection, nivolumab, 1 mg (deprecated 2015) (UMLS:HCPCS:C9453); or
Injection, ipilimumab, 1 mg (UMLS:HCPCS:J9228); or
Injection, atezolizumab, 10 mg (UMLS:HCPCS:J9022); or
Injection, atezolizumab, 10 mg (deprecated 2017) (UMLS:HCPCS:C9483); or
Injection, avelumab, 10 mg (UMLS:HCPCS:J9023); or
Injection, avelumab, 10 mg (deprecated 2017) (UMLS:HCPCS:C9491); or
Injection, durvalumab, 10 mg (UMLS:HCPCS:J9173); or
Injection, cemiplimab-rwlc, 1 mg (UMLS:HCPCS:J9119); or
Injection, durvalumab, 10 mg (deprecated 2018) (UMLS:HCPCS:C9492); or
Injection, cemiplimab-rwlc, 1 mg (deprecated 2019) (UMLS:HCPCS:C9044).
Patients cannot have:
any of the following:
ceritinib (NLM:RXNORM:1535457); or
afatinib (NLM:RXNORM:1430438); or
alectinib (NLM:RXNORM:1727455); or
crizotinib (NLM:RXNORM:1148495); or
brigatinib (NLM:RXNORM:1921217); or
erlotinib (NLM:RXNORM:337525); or
dabrafenib (NLM:RXNORM:1424911); or
gefitinib (NLM:RXNORM:328134); or
osimertinib (NLM:RXNORM:1721560); or
trametinib (NLM:RXNORM:1425099).
NSCLC DX: Any instance of NSCLC DX occurred within 6 months on or before any instance of ICI
Patients must have:
any of the following:
Malignant neoplasm of bronchus and lung (UMLS:ICD10CM:C34); or
Bronchus and lung (UMLS:ICDO3:C34); or
Malignant neoplasm of bronchus and lung (UMLS:ICD10CM:C34); or
Stage 1 (TNX:STAGE:S1); or
Stage 2 (TNX:STAGE:S2); or
Stage 3 (TNX:STAGE:S3); or
Non-small cell carcinoma (TNX:TNX:NSCC); or
Malignant neoplasm of bronchus and lung (UMLS:ICD10CM:C34); or
CD274 (GENE:17635); or
Cells.programmed cell death ligand 1/100 viable tumor cells in Tissue by Immune stain (UMLS:LNC:83053-9).

Patients cannot have:
any of the following:
Small cell carcinoma (TNX:TNX:SCC); or
Small cell carcinoma, NOS (UMLS:ICDO3:8041/3); or
Combined small cell carcinoma (UMLS:ICDO3:8045/3); or
Small cell carcinoma, fusiform cell (UMLS:ICDO3:8043/3); or
Small cell carcinoma, intermediate cell (UMLS:ICDO3:8044/3); or
EGFR (GENE:3236); or
ALK (GENE:427).

CR: The terms in this group occurred at any time
Patients must have:
any of the following:
Stereotactic radiosurgery (particle beam, gamma ray, or linear accelerator); 1 simple cranial lesion (UMLS:CPT:61796); or
Stereotactic radiosurgery (particle beam, gamma ray, or linear accelerator); 1 complex cranial lesion (UMLS:CPT:61798); or
Stereotactic radiosurgery (particle beam, gamma ray, or linear accelerator); each additional cranial lesion, complex (List separately in addition to code for primary procedure) (UMLS:CPT:61799); or
Application of stereotactic headframe for stereotactic radiosurgery (List separately in addition to code for primary procedure) (UMLS:CPT:61800); or
Radiation treatment delivery, stereotactic radiosurgery (SRS), complete course of treatment of cranial lesion(s) consisting of 1 session; multi-source Cobalt 60 based (UMLS:CPT:77371); or
Radiation treatment delivery, stereotactic radiosurgery (SRS), complete course of treatment of cranial lesion(s) consisting of 1 session; linear accelerator based (UMLS:CPT:77372); or
Stereotactic radiation treatment management of cranial lesion(s) (complete course of treatment consisting of 1 session) (UMLS:CPT:77432); or
Image-guided robotic linear accelerator-based stereotactic radiosurgery, complete course of therapy in one session or first session of fractionated treatment (UMLS:HCPCS:G0339); or
Image-guided robotic linear accelerator-based stereotactic radiosurgery, delivery including collimator changes and custom plugging, fractionated treatment, all lesions, per session, second through fifth sessions, maximum five sessions per course of treatment (UMLS:HCPCS:G0340); or
Radiation treatment delivery, superficial and/or ortho voltage, per day (UMLS:CPT:77401); or
Radiation treatment delivery, >=1 MeV; simple (UMLS:CPT:77402); or
Radiation treatment delivery, >=1 MeV; intermediate (UMLS:CPT:77407); or
Radiation treatment delivery, >=1 MeV; complex (UMLS:CPT:77412); or
Radiation treatment delivery, single treatment area,single port or parallel opposed ports, simple blocks or no blocks: 6-10 mev (UMLS:HCPCS:G6004); or
Radiation treatment delivery, single treatment area,single port or parallel opposed ports, simple blocks or no blocks: 11-19 mev (UMLS:HCPCS:G6005); or
Radiation treatment delivery, single treatment area,single port or parallel opposed ports, simple blocks or no blocks: 20 mev or greater (UMLS:HCPCS:G6006); or
Radiation treatment delivery, 2 separate treatment areas, 3 or more ports on a single treatment area, use of multiple blocks: up to 5 mev (UMLS:HCPCS:G6007); or
Radiation treatment delivery, 2 separate treatment areas, 3 or more ports on a single treatment area, use of multiple blocks: 6-10 mev (UMLS:HCPCS:G6008); or
Radiation treatment delivery, 2 separate treatment areas, 3 or more ports on a single treatment area,

use of multiple blocks: 11-19 mev (UMLS:HCPCS:G6009); or
Radiation treatment delivery, 2 separate treatment areas, 3 or more ports on a single treatment area, use of multiple blocks: 20 mev or greater (UMLS:HCPCS:G6010); or
Radiation treatment delivery,3 or more separate treatment areas, custom blocking, tangential ports, wedges, rotational beam, compensators, electron beam; up to 5 mev (UMLS:HCPCS:G6011); or
Radiation treatment delivery,3 or more separate treatment areas, custom blocking, tangential ports, wedges, rotational beam, compensators, electron beam; 6-10 mev (UMLS:HCPCS:G6012); or
Radiation treatment delivery,3 or more separate treatment areas, custom blocking, tangential ports, wedges, rotational beam, compensators, electron beam; 11-19 mev (UMLS:HCPCS:G6013); or
Radiation treatment delivery,3 or more separate treatment areas, custom blocking, tangential ports, wedges, rotational beam, compensators, electron beam; 20 mev or greater (UMLS:HCPCS:G6014); or
Compensator-based beam modulation treatment delivery of inverse planned treatment using 3 or more high resolution (milled or cast) compensator, convergent beam modulated fields, per treatment session (UMLS:HCPCS:G6016); or
Stereotactic body radiation therapy, treatment delivery, per fraction to 1 or more lesions, including image guidance, entire course not to exceed 5 fractions (UMLS:CPT:77373).
NSCLC DX: Any instance of NSCLC DX occurred within 6 months on or before any instance of CR
Patients must have:
any of the following:
Malignant neoplasm of bronchus and lung (UMLS:ICD10CM:C34); or
Bronchus and lung (UMLS:ICDO3:C34); or
Malignant neoplasm of bronchus and lung (UMLS:ICD10CM:C34); or
Stage 1 (TNX:STAGE:S1); or
Stage 2 (TNX:STAGE:S2); or
Stage 3 (TNX:STAGE:S3); or
Non-small cell carcinoma (TNX:TNX:NSCC); or
Malignant neoplasm of bronchus and lung (UMLS:ICD10CM:C34); or
CD274 (GENE:17635); or
Cells.programmed cell death ligand 1/100 viable tumor cells in Tissue by Immune stain (UMLS:LNC:83053-9).
Patients cannot have:
any of the following:
Small cell carcinoma (TNX:TNX:SCC); or
Small cell carcinoma, NOS (UMLS:ICDO3:8041/3); or
Combined small cell carcinoma (UMLS:ICDO3:8045/3); or
Small cell carcinoma, fusiform cell (UMLS:ICDO3:8043/3); or
Small cell carcinoma, intermediate cell (UMLS:ICDO3:8044/3); or
EGFR (GENE:3236); or
ALK (GENE:427).

ICI: The terms in this group occurred at any time
Patients must have:
any of the following:
Injection, pembrolizumab, 1 mg (UMLS:HCPCS:J9271); or
Injection, pembrolizumab, 1 mg (deprecated 2015) (UMLS:HCPCS:C9027); or
Injection, nivolumab, 1 mg (UMLS:HCPCS:J9299); or
Injection, nivolumab and relatlimab-rmbw, 3 mg/1 mg (UMLS:HCPCS:J9298); or

Injection, nivolumab, 1 mg (deprecated 2015) (UMLS:HCPCS:C9453); or
Injection, ipilimumab, 1 mg (UMLS:HCPCS:J9228); or
Injection, atezolizumab, 10 mg (UMLS:HCPCS:J9022); or
Injection, atezolizumab, 10 mg (deprecated 2017) (UMLS:HCPCS:C9483); or
Injection, avelumab, 10 mg (UMLS:HCPCS:J9023); or
Injection, avelumab, 10 mg (deprecated 2017) (UMLS:HCPCS:C9491); or
Injection, durvalumab, 10 mg (UMLS:HCPCS:J9173); or
Injection, cemiplimab-rwlc, 1 mg (UMLS:HCPCS:J9119); or
Injection, durvalumab, 10 mg (deprecated 2018) (UMLS:HCPCS:C9492); or
Injection, cemiplimab-rwlc, 1 mg (deprecated 2019) (UMLS:HCPCS:C9044).

CR: Any instance of CR occurred within 15 days before or up to 15 days after any instance of ICI

Patients must have:

any of the following:

Stereotactic radiosurgery (particle beam, gamma ray, or linear accelerator); 1 simple cranial lesion (UMLS:CPT:61796); or

Stereotactic radiosurgery (particle beam, gamma ray, or linear accelerator); 1 complex cranial lesion (UMLS:CPT:61798); or

Stereotactic radiosurgery (particle beam, gamma ray, or linear accelerator); each additional cranial lesion, complex (List separately in addition to code for primary procedure) (UMLS:CPT:61799); or

Application of stereotactic headframe for stereotactic radiosurgery (List separately in addition to code for primary procedure) (UMLS:CPT:61800); or

Radiation treatment delivery, stereotactic radiosurgery (SRS), complete course of treatment of cranial lesion(s) consisting of 1 session; multi-source Cobalt 60 based (UMLS:CPT:77371); or

Radiation treatment delivery, stereotactic radiosurgery (SRS), complete course of treatment of cranial lesion(s) consisting of 1 session; linear accelerator based (UMLS:CPT:77372); or

Stereotactic radiation treatment management of cranial lesion(s) (complete course of treatment consisting of 1 session) (UMLS:CPT:77432); or

Image-guided robotic linear accelerator-based stereotactic radiosurgery, complete course of therapy in one session or first session of fractionated treatment (UMLS:HCPCS:G0339); or

Image-guided robotic linear accelerator-based stereotactic radiosurgery, delivery including collimator changes and custom plugging, fractionated treatment, all lesions, per session, second through fifth sessions, maximum five sessions per course of treatment (UMLS:HCPCS:G0340); or

Radiation treatment delivery, superficial and/or ortho voltage, per day (UMLS:CPT:77401); or

Radiation treatment delivery, >=1 MeV; simple (UMLS:CPT:77402); or

Radiation treatment delivery, >=1 MeV; intermediate (UMLS:CPT:77407); or

Radiation treatment delivery, >=1 MeV; complex (UMLS:CPT:77412); or

Radiation treatment delivery, single treatment area,single port or parallel opposed ports, simple blocks or no blocks: 6-10 mev (UMLS:HCPCS:G6004); or

Radiation treatment delivery, single treatment area,single port or parallel opposed ports, simple blocks or no blocks: 11-19 mev (UMLS:HCPCS:G6005); or

Radiation treatment delivery, single treatment area,single port or parallel opposed ports, simple blocks or no blocks: 20 mev or greater (UMLS:HCPCS:G6006); or

Radiation treatment delivery, 2 separate treatment areas, 3 or more ports on a single treatment area, use of multiple blocks: up to 5 mev (UMLS:HCPCS:G6007); or

Radiation treatment delivery, 2 separate treatment areas, 3 or more ports on a single treatment area, use of multiple blocks: 6-10 mev (UMLS:HCPCS:G6008); or

Radiation treatment delivery, 2 separate treatment areas, 3 or more ports on a single treatment area, use of multiple blocks: 11-19 mev (UMLS:HCPCS:G6009); or

Radiation treatment delivery, 2 separate treatment areas, 3 or more ports on a single treatment area, use of multiple blocks: 20 mev or greater (UMLS:HCPCS:G6010); or

Radiation treatment delivery,3 or more separate treatment areas, custom blocking, tangential ports, wedges, rotational beam, compensators, electron beam; up to 5 mev (UMLS:HCPCS:G6011); or

Radiation treatment delivery,3 or more separate treatment areas, custom blocking, tangential ports, wedges, rotational beam, compensators, electron beam; 6-10 mev (UMLS:HCPCS:G6012); or

Radiation treatment delivery,3 or more separate treatment areas, custom blocking, tangential ports, wedges, rotational beam, compensators, electron beam; 11-19 mev (UMLS:HCPCS:G6013); or

Radiation treatment delivery,3 or more separate treatment areas, custom blocking, tangential ports, wedges, rotational beam, compensators, electron beam; 20 mev or greater (UMLS:HCPCS:G6014); or

Compensator-based beam modulation treatment delivery of inverse planned treatment using 3 or more high resolution (milled or cast) compensator, convergent beam modulated fields, per treatment session (UMLS:HCPCS:G6016); or

Stereotactic body radiation therapy, treatment delivery, per fraction to 1 or more lesions, including image guidance, entire course not to exceed 5 fractions (UMLS:CPT:77373).

Query Criteria for Cohort 2 (query name: ICI-CR 31+days new)

Patients must have:
Age (Age) (between 65 and 120 years (most recent occurrence)).

All the following must be satisfied:

NSCLC DX: The terms in this group occurred at any time
Patients must have:
any of the following:
Malignant neoplasm of bronchus and lung (UMLS:ICD10CM:C34); or
Bronchus and lung (UMLS:ICDO3:C34); or
Malignant neoplasm of bronchus and lung (UMLS:ICD10CM:C34); or
Stage 1 (TNX:STAGE:S1); or
Stage 2 (TNX:STAGE:S2); or
Stage 3 (TNX:STAGE:S3); or
Non-small cell carcinoma (TNX:TNX:NSCC); or
Malignant neoplasm of bronchus and lung (UMLS:ICD10CM:C34); or
CD274 (GENE:17635); or
Cells.programmed cell death ligand 1/100 viable tumor cells in Tissue by Immune stain (UMLS:LNC:83053-9).
Patients cannot have:
any of the following:
Small cell carcinoma (TNX:TNX:SCC); or
Small cell carcinoma, NOS (UMLS:ICDO3:8041/3); or
Combined small cell carcinoma (UMLS:ICDO3:8045/3); or
Small cell carcinoma, fusiform cell (UMLS:ICDO3:8043/3); or
Small cell carcinoma, intermediate cell (UMLS:ICDO3:8044/3); or
EGFR (GENE:3236); or
ALK (GENE:427).
BM DX: Any instance of BM DX occurred within 1 day before or up to 1 day after any instance of NSCLC DX
Patients must have:
any of the following:
Secondary malignant neoplasm of brain (UMLS:ICD10CM:C79.31); or
Stage 4 (TNX:STAGE:S4); or
M1 (TNX:STAGE:TNM_M1).

ICI: The terms in this group occurred at any time
Patients must have:
any of the following:
Injection, pembrolizumab, 1 mg (UMLS:HCPCS:J9271); or
Injection, pembrolizumab, 1 mg (deprecated 2015) (UMLS:HCPCS:C9027); or
Injection, nivolumab, 1 mg (UMLS:HCPCS:J9299); or
Injection, nivolumab and relatlimab-rmbw, 3 mg/1 mg (UMLS:HCPCS:J9298); or
Injection, nivolumab, 1 mg (deprecated 2015) (UMLS:HCPCS:C9453); or
Injection, ipilimumab, 1 mg (UMLS:HCPCS:J9228); or
Injection, atezolizumab, 10 mg (UMLS:HCPCS:J9022); or
Injection, atezolizumab, 10 mg (deprecated 2017) (UMLS:HCPCS:C9483); or
Injection, avelumab, 10 mg (UMLS:HCPCS:J9023); or
Injection, avelumab, 10 mg (deprecated 2017) (UMLS:HCPCS:C9491); or
Injection, durvalumab, 10 mg (UMLS:HCPCS:J9173); or
Injection, cemiplimab-rwlc, 1 mg (UMLS:HCPCS:J9119); or
Injection, durvalumab, 10 mg (deprecated 2018) (UMLS:HCPCS:C9492); or
Injection, cemiplimab-rwlc, 1 mg (deprecated 2019) (UMLS:HCPCS:C9044).
Patients cannot have:
any of the following:
ceritinib (NLM:RXNORM:1535457); or
afatinib (NLM:RXNORM:1430438); or
alectinib (NLM:RXNORM:1727455); or
crizotinib (NLM:RXNORM:1148495); or
brigatinib (NLM:RXNORM:1921217); or
erlotinib (NLM:RXNORM:337525); or
dabrafenib (NLM:RXNORM:1424911); or
gefitinib (NLM:RXNORM:328134); or
osimertinib (NLM:RXNORM:1721560); or
trametinib (NLM:RXNORM:1425099).
NSCLC DX: Any instance of NSCLC DX occurred within 6 months on or before any instance of ICI
Patients must have:
any of the following:
Malignant neoplasm of bronchus and lung (UMLS:ICD10CM:C34); or
Bronchus and lung (UMLS:ICDO3:C34); or
Malignant neoplasm of bronchus and lung (UMLS:ICD10CM:C34); or
Stage 1 (TNX:STAGE:S1); or
Stage 2 (TNX:STAGE:S2); or
Stage 3 (TNX:STAGE:S3); or
Non-small cell carcinoma (TNX:TNX:NSCC); or
Malignant neoplasm of bronchus and lung (UMLS:ICD10CM:C34); or
CD274 (GENE:17635); or
Cells.programmed cell death ligand 1/100 viable tumor cells in Tissue by Immune stain (UMLS:LNC:83053-9).
Patients cannot have:
any of the following:
Small cell carcinoma (TNX:TNX:SCC); or

Small cell carcinoma, NOS (UMLS:ICDO3:8041/3); or
Combined small cell carcinoma (UMLS:ICDO3:8045/3); or
Small cell carcinoma, fusiform cell (UMLS:ICDO3:8043/3); or
Small cell carcinoma, intermediate cell (UMLS:ICDO3:8044/3); or
EGFR (GENE:3236); or
ALK (GENE:427).

CR: The terms in this group occurred at any time
Patients must have:
any of the following:
Stereotactic radiosurgery (particle beam, gamma ray, or linear accelerator); 1 simple cranial lesion (UMLS:CPT:61796); or
Stereotactic radiosurgery (particle beam, gamma ray, or linear accelerator); 1 complex cranial lesion (UMLS:CPT:61798); or
Stereotactic radiosurgery (particle beam, gamma ray, or linear accelerator); each additional cranial lesion, complex (List separately in addition to code for primary procedure) (UMLS:CPT:61799); or
Application of stereotactic headframe for stereotactic radiosurgery (List separately in addition to code for primary procedure) (UMLS:CPT:61800); or
Radiation treatment delivery, stereotactic radiosurgery (SRS), complete course of treatment of cranial lesion(s) consisting of 1 session; multi-source Cobalt 60 based (UMLS:CPT:77371); or
Radiation treatment delivery, stereotactic radiosurgery (SRS), complete course of treatment of cranial lesion(s) consisting of 1 session; linear accelerator based (UMLS:CPT:77372); or
Stereotactic radiation treatment management of cranial lesion(s) (complete course of treatment consisting of 1 session) (UMLS:CPT:77432); or
Image-guided robotic linear accelerator-based stereotactic radiosurgery, complete course of therapy in one session or first session of fractionated treatment (UMLS:HCPCS:G0339); or
Image-guided robotic linear accelerator-based stereotactic radiosurgery, delivery including collimator changes and custom plugging, fractionated treatment, all lesions, per session, second through fifth sessions, maximum five sessions per course of treatment (UMLS:HCPCS:G0340); or
Radiation treatment delivery, superficial and/or ortho voltage, per day (UMLS:CPT:77401); or
Radiation treatment delivery, >=1 MeV; simple (UMLS:CPT:77402); or
Radiation treatment delivery, >=1 MeV; intermediate (UMLS:CPT:77407); or
Radiation treatment delivery, >=1 MeV; complex (UMLS:CPT:77412); or
Radiation treatment delivery, single treatment area,single port or parallel opposed ports, simple blocks or no blocks: 6-10 mev (UMLS:HCPCS:G6004); or
Radiation treatment delivery, single treatment area,single port or parallel opposed ports, simple blocks or no blocks: 11-19 mev (UMLS:HCPCS:G6005); or
Radiation treatment delivery, single treatment area,single port or parallel opposed ports, simple blocks or no blocks: 20 mev or greater (UMLS:HCPCS:G6006); or
Radiation treatment delivery, 2 separate treatment areas, 3 or more ports on a single treatment area, use of multiple blocks: up to 5 mev (UMLS:HCPCS:G6007); or
Radiation treatment delivery, 2 separate treatment areas, 3 or more ports on a single treatment area, use of multiple blocks: 6-10 mev (UMLS:HCPCS:G6008); or
Radiation treatment delivery, 2 separate treatment areas, 3 or more ports on a single treatment area, use of multiple blocks: 11-19 mev (UMLS:HCPCS:G6009); or
Radiation treatment delivery, 2 separate treatment areas, 3 or more ports on a single treatment area, use of multiple blocks: 20 mev or greater (UMLS:HCPCS:G6010); or

Radiation treatment delivery,3 or more separate treatment areas, custom blocking, tangential ports, wedges, rotational beam, compensators, electron beam; up to 5 mev (UMLS:HCPCS:G6011); or

Radiation treatment delivery,3 or more separate treatment areas, custom blocking, tangential ports, wedges, rotational beam, compensators, electron beam; 6-10 mev (UMLS:HCPCS:G6012); or

Radiation treatment delivery,3 or more separate treatment areas, custom blocking, tangential ports, wedges, rotational beam, compensators, electron beam; 11-19 mev (UMLS:HCPCS:G6013); or

Radiation treatment delivery,3 or more separate treatment areas, custom blocking, tangential ports, wedges, rotational beam, compensators, electron beam; 20 mev or greater (UMLS:HCPCS:G6014); or

Compensator-based beam modulation treatment delivery of inverse planned treatment using 3 or more high resolution (milled or cast) compensator, convergent beam modulated fields, per treatment session (UMLS:HCPCS:G6016); or

Stereotactic body radiation therapy, treatment delivery, per fraction to 1 or more lesions, including image guidance, entire course not to exceed 5 fractions (UMLS:CPT:77373).

NSCLC DX: Any instance of NSCLC DX occurred within 6 months on or before any instance of CR

Patients must have:

any of the following:

Malignant neoplasm of bronchus and lung (UMLS:ICD10CM:C34); or
Bronchus and lung (UMLS:ICDO3:C34); or
Malignant neoplasm of bronchus and lung (UMLS:ICD10CM:C34); or
Stage 1 (TNX:STAGE:S1); or
Stage 2 (TNX:STAGE:S2); or
Stage 3 (TNX:STAGE:S3); or
Non-small cell carcinoma (TNX:TNX:NSCC); or
Malignant neoplasm of bronchus and lung (UMLS:ICD10CM:C34); or
CD274 (GENE:17635); or
Cells.programmed cell death ligand 1/100 viable tumor cells in Tissue by Immune stain (UMLS:LNC:83053-9).

Patients cannot have:

any of the following:

Small cell carcinoma (TNX:TNX:SCC); or
Small cell carcinoma, NOS (UMLS:ICDO3:8041/3); or
Combined small cell carcinoma (UMLS:ICDO3:8045/3); or
Small cell carcinoma, fusiform cell (UMLS:ICDO3:8043/3); or
Small cell carcinoma, intermediate cell (UMLS:ICDO3:8044/3); or
EGFR (GENE:3236); or
ALK (GENE:427).

Any of the following must be satisfied:

ICI: The terms in this group occurred at any time

Patients must have:

any of the following:

Injection, pembrolizumab, 1 mg (UMLS:HCPCS:J9271); or
Injection, pembrolizumab, 1 mg (deprecated 2015) (UMLS:HCPCS:C9027); or
Injection, nivolumab, 1 mg (UMLS:HCPCS:J9299); or
Injection, nivolumab and relatlimab-rmbw, 3 mg/1 mg (UMLS:HCPCS:J9298); or
Injection, nivolumab, 1 mg (deprecated 2015) (UMLS:HCPCS:C9453); or

Injection, ipilimumab, 1 mg (UMLS:HCPCS:J9228); or
Injection, atezolizumab, 10 mg (UMLS:HCPCS:J9022); or
Injection, atezolizumab, 10 mg (deprecated 2017) (UMLS:HCPCS:C9483); or
Injection, avelumab, 10 mg (UMLS:HCPCS:J9023); or
Injection, avelumab, 10 mg (deprecated 2017) (UMLS:HCPCS:C9491); or
Injection, durvalumab, 10 mg (UMLS:HCPCS:J9173); or
Injection, cemiplimab-rwlc, 1 mg (UMLS:HCPCS:J9119); or
Injection, durvalumab, 10 mg (deprecated 2018) (UMLS:HCPCS:C9492); or
Injection, cemiplimab-rwlc, 1 mg (deprecated 2019) (UMLS:HCPCS:C9044).

CR: Any instance of CR occurred within 31 days and 6 months after any instance of ICI

Patients must have:

any of the following:

Stereotactic radiosurgery (particle beam, gamma ray, or linear accelerator); 1 simple cranial lesion (UMLS:CPT:61796); or

Stereotactic radiosurgery (particle beam, gamma ray, or linear accelerator); 1 complex cranial lesion (UMLS:CPT:61798); or

Stereotactic radiosurgery (particle beam, gamma ray, or linear accelerator); each additional cranial lesion, complex (List separately in addition to code for primary procedure) (UMLS:CPT:61799); or

Application of stereotactic headframe for stereotactic radiosurgery (List separately in addition to code for primary procedure) (UMLS:CPT:61800); or

Radiation treatment delivery, stereotactic radiosurgery (SRS), complete course of treatment of cranial lesion(s) consisting of 1 session; multi-source Cobalt 60 based (UMLS:CPT:77371); or

Radiation treatment delivery, stereotactic radiosurgery (SRS), complete course of treatment of cranial lesion(s) consisting of 1 session; linear accelerator based (UMLS:CPT:77372); or

Stereotactic radiation treatment management of cranial lesion(s) (complete course of treatment consisting of 1 session) (UMLS:CPT:77432); or

Image-guided robotic linear accelerator-based stereotactic radiosurgery, complete course of therapy in one session or first session of fractionated treatment (UMLS:HCPCS:G0339); or

Image-guided robotic linear accelerator-based stereotactic radiosurgery, delivery including collimator changes and custom plugging, fractionated treatment, all lesions, per session, second through fifth sessions, maximum five sessions per course of treatment (UMLS:HCPCS:G0340); or

Radiation treatment delivery, superficial and/or ortho voltage, per day (UMLS:CPT:77401); or

Radiation treatment delivery, >=1 MeV; simple (UMLS:CPT:77402); or

Radiation treatment delivery, >=1 MeV; intermediate (UMLS:CPT:77407); or

Radiation treatment delivery, >=1 MeV; complex (UMLS:CPT:77412); or

Radiation treatment delivery, single treatment area,single port or parallel opposed ports, simple blocks or no blocks: 6-10 mev (UMLS:HCPCS:G6004); or

Radiation treatment delivery, single treatment area,single port or parallel opposed ports, simple blocks or no blocks: 11-19 mev (UMLS:HCPCS:G6005); or

Radiation treatment delivery, single treatment area,single port or parallel opposed ports, simple blocks or no blocks: 20 mev or greater (UMLS:HCPCS:G6006); or

Radiation treatment delivery, 2 separate treatment areas, 3 or more ports on a single treatment area, use of multiple blocks: up to 5 mev (UMLS:HCPCS:G6007); or

Radiation treatment delivery, 2 separate treatment areas, 3 or more ports on a single treatment area, use of multiple blocks: 6-10 mev (UMLS:HCPCS:G6008); or

Radiation treatment delivery, 2 separate treatment areas, 3 or more ports on a single treatment area, use of multiple blocks: 11-19 mev (UMLS:HCPCS:G6009); or

Radiation treatment delivery, 2 separate treatment areas, 3 or more ports on a single treatment area, use of multiple blocks: 20 mev or greater (UMLS:HCPCS:G6010); or

Radiation treatment delivery,3 or more separate treatment areas, custom blocking, tangential ports,

wedges, rotational beam, compensators, electron beam; up to 5 mev (UMLS:HCPCS:G6011); or
Radiation treatment delivery,3 or more separate treatment areas, custom blocking, tangential ports, wedges, rotational beam, compensators, electron beam; 6-10 mev (UMLS:HCPCS:G6012); or
Radiation treatment delivery,3 or more separate treatment areas, custom blocking, tangential ports, wedges, rotational beam, compensators, electron beam; 11-19 mev (UMLS:HCPCS:G6013); or
Radiation treatment delivery,3 or more separate treatment areas, custom blocking, tangential ports, wedges, rotational beam, compensators, electron beam; 20 mev or greater (UMLS:HCPCS:G6014); or
Compensator-based beam modulation treatment delivery of inverse planned treatment using 3 or more high resolution (milled or cast) compensator, convergent beam modulated fields, per treatment session (UMLS:HCPCS:G6016); or
Stereotactic body radiation therapy, treatment delivery, per fraction to 1 or more lesions, including image guidance, entire course not to exceed 5 fractions (UMLS:CPT:77373).

ICI: The terms in this group occurred at any time
Patients must have:
any of the following:
Injection, pembrolizumab, 1 mg (UMLS:HCPCS:J9271); or
Injection, pembrolizumab, 1 mg (deprecated 2015) (UMLS:HCPCS:C9027); or
Injection, nivolumab, 1 mg (UMLS:HCPCS:J9299); or
Injection, nivolumab and relatlimab-rmbw, 3 mg/1 mg (UMLS:HCPCS:J9298); or
Injection, nivolumab, 1 mg (deprecated 2015) (UMLS:HCPCS:C9453); or
Injection, ipilimumab, 1 mg (UMLS:HCPCS:J9228); or
Injection, atezolizumab, 10 mg (UMLS:HCPCS:J9022); or
Injection, atezolizumab, 10 mg (deprecated 2017) (UMLS:HCPCS:C9483); or
Injection, avelumab, 10 mg (UMLS:HCPCS:J9023); or
Injection, avelumab, 10 mg (deprecated 2017) (UMLS:HCPCS:C9491); or
Injection, durvalumab, 10 mg (UMLS:HCPCS:J9173); or
Injection, cemiplimab-rwlc, 1 mg (UMLS:HCPCS:J9119); or
Injection, durvalumab, 10 mg (deprecated 2018) (UMLS:HCPCS:C9492); or
Injection, cemiplimab-rwlc, 1 mg (deprecated 2019) (UMLS:HCPCS:C9044).

CR: Any instance of CR occurred within 6 months and 31 days before any instance of ICI
Patients must have:
any of the following:
Stereotactic radiosurgery (particle beam, gamma ray, or linear accelerator); 1 simple cranial lesion (UMLS:CPT:61796); or
Stereotactic radiosurgery (particle beam, gamma ray, or linear accelerator); 1 complex cranial lesion (UMLS:CPT:61798); or
Stereotactic radiosurgery (particle beam, gamma ray, or linear accelerator); each additional cranial lesion, complex (List separately in addition to code for primary procedure) (UMLS:CPT:61799); or
Application of stereotactic headframe for stereotactic radiosurgery (List separately in addition to code for primary procedure) (UMLS:CPT:61800); or
Radiation treatment delivery, stereotactic radiosurgery (SRS), complete course of treatment of cranial lesion(s) consisting of 1 session; multi-source Cobalt 60 based (UMLS:CPT:77371); or
Radiation treatment delivery, stereotactic radiosurgery (SRS), complete course of treatment of cranial lesion(s) consisting of 1 session; linear accelerator based (UMLS:CPT:77372); or
Stereotactic radiation treatment management of cranial lesion(s) (complete course of treatment consisting of 1 session) (UMLS:CPT:77432); or
Image-guided robotic linear accelerator-based stereotactic radiosurgery, complete course of therapy in one session or first session of fractionated treatment (UMLS:HCPCS:G0339); or

Image-guided robotic linear accelerator-based stereotactic radiosurgery, delivery including collimator changes and custom plugging, fractionated treatment, all lesions, per session, second through fifth sessions, maximum five sessions per course of treatment (UMLS:HCPCS:G0340); or
Radiation treatment delivery, superficial and/or ortho voltage, per day (UMLS:CPT:77401); or
Radiation treatment delivery, >=1 MeV; simple (UMLS:CPT:77402); or
Radiation treatment delivery, >=1 MeV; intermediate (UMLS:CPT:77407); or
Radiation treatment delivery, >=1 MeV; complex (UMLS:CPT:77412); or
Radiation treatment delivery, single treatment area,single port or parallel opposed ports, simple blocks or no blocks: 6-10 mev (UMLS:HCPCS:G6004); or
Radiation treatment delivery, single treatment area,single port or parallel opposed ports, simple blocks or no blocks: 11-19 mev (UMLS:HCPCS:G6005); or
Radiation treatment delivery, single treatment area,single port or parallel opposed ports, simple blocks or no blocks: 20 mev or greater (UMLS:HCPCS:G6006); or
Radiation treatment delivery, 2 separate treatment areas, 3 or more ports on a single treatment area, use of multiple blocks: up to 5 mev (UMLS:HCPCS:G6007); or
Radiation treatment delivery, 2 separate treatment areas, 3 or more ports on a single treatment area, use of multiple blocks: 6-10 mev (UMLS:HCPCS:G6008); or
Radiation treatment delivery, 2 separate treatment areas, 3 or more ports on a single treatment area, use of multiple blocks: 11-19 mev (UMLS:HCPCS:G6009); or
Radiation treatment delivery, 2 separate treatment areas, 3 or more ports on a single treatment area, use of multiple blocks: 20 mev or greater (UMLS:HCPCS:G6010); or
Radiation treatment delivery,3 or more separate treatment areas, custom blocking, tangential ports, wedges, rotational beam, compensators, electron beam; up to 5 mev (UMLS:HCPCS:G6011); or
Radiation treatment delivery,3 or more separate treatment areas, custom blocking, tangential ports, wedges, rotational beam, compensators, electron beam; 6-10 mev (UMLS:HCPCS:G6012); or
Radiation treatment delivery,3 or more separate treatment areas, custom blocking, tangential ports, wedges, rotational beam, compensators, electron beam; 11-19 mev (UMLS:HCPCS:G6013); or
Radiation treatment delivery,3 or more separate treatment areas, custom blocking, tangential ports, wedges, rotational beam, compensators, electron beam; 20 mev or greater (UMLS:HCPCS:G6014); or
Compensator-based beam modulation treatment delivery of inverse planned treatment using 3 or more high resolution (milled or cast) compensator, convergent beam modulated fields, per treatment session (UMLS:HCPCS:G6016); or
Stereotactic body radiation therapy, treatment delivery, per fraction to 1 or more lesions, including image guidance, entire course not to exceed 5 fractions (UMLS:CPT:77373).

Appendix B – Text Representation of the Analysis Setup

This section contains the Index Event definition for each cohort.

The index event for Cohort 1 (query name: ICI-CR 0-15 days) is defined as the following:

All the following must be satisfied:

ICI: The terms in this group occurred at any time
Patients must have:
any of the following:
Injection, pembrolizumab, 1 mg (UMLS:HCPCS:J9271); or
Injection, pembrolizumab, 1 mg (deprecated 2015) (UMLS:HCPCS:C9027); or
Injection, nivolumab, 1 mg (UMLS:HCPCS:J9299); or
Injection, nivolumab and relatlimab-rmbw, 3 mg/1 mg (UMLS:HCPCS:J9298); or

Injection, nivolumab, 1 mg (deprecated 2015) (UMLS:HCPCS:C9453); or
Injection, ipilimumab, 1 mg (UMLS:HCPCS:J9228); or
Injection, atezolizumab, 10 mg (UMLS:HCPCS:J9022); or
Injection, atezolizumab, 10 mg (deprecated 2017) (UMLS:HCPCS:C9483); or
Injection, avelumab, 10 mg (UMLS:HCPCS:J9023); or
Injection, avelumab, 10 mg (deprecated 2017) (UMLS:HCPCS:C9491); or
Injection, durvalumab, 10 mg (UMLS:HCPCS:J9173); or
Injection, cemiplimab-rwlc, 1 mg (UMLS:HCPCS:J9119); or
Injection, durvalumab, 10 mg (deprecated 2018) (UMLS:HCPCS:C9492); or
Injection, cemiplimab-rwlc, 1 mg (deprecated 2019) (UMLS:HCPCS:C9044).

CR: Any instance of CR occurred within 15 days before or up to 15 days after any instance of ICI

Patients must have:

any of the following:

Stereotactic radiosurgery (particle beam, gamma ray, or linear accelerator); 1 simple cranial lesion (UMLS:CPT:61796); or

Stereotactic radiosurgery (particle beam, gamma ray, or linear accelerator); 1 complex cranial lesion (UMLS:CPT:61798); or

Stereotactic radiosurgery (particle beam, gamma ray, or linear accelerator); each additional cranial lesion, complex (List separately in addition to code for primary procedure) (UMLS:CPT:61799); or

Application of stereotactic headframe for stereotactic radiosurgery (List separately in addition to code for primary procedure) (UMLS:CPT:61800); or

Radiation treatment delivery, stereotactic radiosurgery (SRS), complete course of treatment of cranial lesion(s) consisting of 1 session; multi-source Cobalt 60 based (UMLS:CPT:77371); or

Radiation treatment delivery, stereotactic radiosurgery (SRS), complete course of treatment of cranial lesion(s) consisting of 1 session; linear accelerator based (UMLS:CPT:77372); or

Stereotactic radiation treatment management of cranial lesion(s) (complete course of treatment consisting of 1 session) (UMLS:CPT:77432); or

Image-guided robotic linear accelerator-based stereotactic radiosurgery, complete course of therapy in one session or first session of fractionated treatment (UMLS:HCPCS:G0339); or

Image-guided robotic linear accelerator-based stereotactic radiosurgery, delivery including collimator changes and custom plugging, fractionated treatment, all lesions, per session, second through fifth sessions, maximum five sessions per course of treatment (UMLS:HCPCS:G0340); or

Radiation treatment delivery, superficial and/or ortho voltage, per day (UMLS:CPT:77401); or

Radiation treatment delivery, >=1 MeV; simple (UMLS:CPT:77402); or

Radiation treatment delivery, >=1 MeV; intermediate (UMLS:CPT:77407); or

Radiation treatment delivery, >=1 MeV; complex (UMLS:CPT:77412); or

Radiation treatment delivery, single treatment area,single port or parallel opposed ports, simple blocks or no blocks: 6-10 mev (UMLS:HCPCS:G6004); or

Radiation treatment delivery, single treatment area,single port or parallel opposed ports, simple blocks or no blocks: 11-19 mev (UMLS:HCPCS:G6005); or

Radiation treatment delivery, single treatment area,single port or parallel opposed ports, simple blocks or no blocks: 20 mev or greater (UMLS:HCPCS:G6006); or

Radiation treatment delivery, 2 separate treatment areas, 3 or more ports on a single treatment area, use of multiple blocks: up to 5 mev (UMLS:HCPCS:G6007); or

Radiation treatment delivery, 2 separate treatment areas, 3 or more ports on a single treatment area, use of multiple blocks: 6-10 mev (UMLS:HCPCS:G6008); or

Radiation treatment delivery, 2 separate treatment areas, 3 or more ports on a single treatment area, use of multiple blocks: 11-19 mev (UMLS:HCPCS:G6009); or

Radiation treatment delivery, 2 separate treatment areas, 3 or more ports on a single treatment area, use of multiple blocks: 20 mev or greater (UMLS:HCPCS:G6010); or

Radiation treatment delivery,3 or more separate treatment areas, custom blocking, tangential ports, wedges, rotational beam, compensators, electron beam; up to 5 mev (UMLS:HCPCS:G6011); or

Radiation treatment delivery,3 or more separate treatment areas, custom blocking, tangential ports, wedges, rotational beam, compensators, electron beam; 6-10 mev (UMLS:HCPCS:G6012); or

Radiation treatment delivery,3 or more separate treatment areas, custom blocking, tangential ports, wedges, rotational beam, compensators, electron beam; 11-19 mev (UMLS:HCPCS:G6013); or

Radiation treatment delivery,3 or more separate treatment areas, custom blocking, tangential ports, wedges, rotational beam, compensators, electron beam; 20 mev or greater (UMLS:HCPCS:G6014); or

Compensator-based beam modulation treatment delivery of inverse planned treatment using 3 or more high resolution (milled or cast) compensator, convergent beam modulated fields, per treatment session (UMLS:HCPCS:G6016); or

Stereotactic body radiation therapy, treatment delivery, per fraction to 1 or more lesions, including image guidance, entire course not to exceed 5 fractions (UMLS:CPT:77373).

The index event for Cohort 2 (query name: ICI-CR 31+days new) is defined as the following:

All the following must be satisfied:

Any of the following must be satisfied:

ICI: The terms in this group occurred at any time
Patients must have:
any of the following:
Injection, pembrolizumab, 1 mg (UMLS:HCPCS:J9271); or
Injection, pembrolizumab, 1 mg (deprecated 2015) (UMLS:HCPCS:C9027); or
Injection, nivolumab, 1 mg (UMLS:HCPCS:J9299); or
Injection, nivolumab and relatlimab-rmbw, 3 mg/1 mg (UMLS:HCPCS:J9298); or
Injection, nivolumab, 1 mg (deprecated 2015) (UMLS:HCPCS:C9453); or
Injection, ipilimumab, 1 mg (UMLS:HCPCS:J9228); or
Injection, atezolizumab, 10 mg (UMLS:HCPCS:J9022); or
Injection, atezolizumab, 10 mg (deprecated 2017) (UMLS:HCPCS:C9483); or
Injection, avelumab, 10 mg (UMLS:HCPCS:J9023); or
Injection, avelumab, 10 mg (deprecated 2017) (UMLS:HCPCS:C9491); or
Injection, durvalumab, 10 mg (UMLS:HCPCS:J9173); or
Injection, cemiplimab-rwlc, 1 mg (UMLS:HCPCS:J9119); or
Injection, durvalumab, 10 mg (deprecated 2018) (UMLS:HCPCS:C9492); or
Injection, cemiplimab-rwlc, 1 mg (deprecated 2019) (UMLS:HCPCS:C9044).

CR: Any instance of CR occurred within 31 days and 6 months after any instance of ICI
Patients must have:
any of the following:

Stereotactic radiosurgery (particle beam, gamma ray, or linear accelerator); 1 simple cranial lesion (UMLS:CPT:61796); or

Stereotactic radiosurgery (particle beam, gamma ray, or linear accelerator); 1 complex cranial lesion (UMLS:CPT:61798); or

Stereotactic radiosurgery (particle beam, gamma ray, or linear accelerator); each additional cranial lesion, complex (List separately in addition to code for primary procedure) (UMLS:CPT:61799); or

Application of stereotactic headframe for stereotactic radiosurgery (List separately in addition to code for primary procedure) (UMLS:CPT:61800); or

Radiation treatment delivery, stereotactic radiosurgery (SRS), complete course of treatment of cranial lesion(s) consisting of 1 session; multi-source Cobalt 60 based (UMLS:CPT:77371); or

Radiation treatment delivery, stereotactic radiosurgery (SRS), complete course of treatment of cranial lesion(s) consisting of 1 session; linear accelerator based (UMLS:CPT:77372); or

Stereotactic radiation treatment management of cranial lesion(s) (complete course of treatment consisting of 1 session) (UMLS:CPT:77432); or

Image-guided robotic linear accelerator-based stereotactic radiosurgery, complete course of therapy in one session or first session of fractionated treatment (UMLS:HCPCS:G0339); or

Image-guided robotic linear accelerator-based stereotactic radiosurgery, delivery including collimator changes and custom plugging, fractionated treatment, all lesions, per session, second through fifth sessions, maximum five sessions per course of treatment (UMLS:HCPCS:G0340); or

Radiation treatment delivery, superficial and/or ortho voltage, per day (UMLS:CPT:77401); or

Radiation treatment delivery, >=1 MeV; simple (UMLS:CPT:77402); or

Radiation treatment delivery, >=1 MeV; intermediate (UMLS:CPT:77407); or

Radiation treatment delivery, >=1 MeV; complex (UMLS:CPT:77412); or

Radiation treatment delivery, single treatment area,single port or parallel opposed ports, simple blocks or no blocks: 6-10 mev (UMLS:HCPCS:G6004); or

Radiation treatment delivery, single treatment area,single port or parallel opposed ports, simple blocks or no blocks: 11-19 mev (UMLS:HCPCS:G6005); or

Radiation treatment delivery, single treatment area,single port or parallel opposed ports, simple blocks or no blocks: 20 mev or greater (UMLS:HCPCS:G6006); or

Radiation treatment delivery, 2 separate treatment areas, 3 or more ports on a single treatment area, use of multiple blocks: up to 5 mev (UMLS:HCPCS:G6007); or

Radiation treatment delivery, 2 separate treatment areas, 3 or more ports on a single treatment area, use of multiple blocks: 6-10 mev (UMLS:HCPCS:G6008); or

Radiation treatment delivery, 2 separate treatment areas, 3 or more ports on a single treatment area, use of multiple blocks: 11-19 mev (UMLS:HCPCS:G6009); or

Radiation treatment delivery, 2 separate treatment areas, 3 or more ports on a single treatment area, use of multiple blocks: 20 mev or greater (UMLS:HCPCS:G6010); or

Radiation treatment delivery,3 or more separate treatment areas, custom blocking, tangential ports, wedges, rotational beam, compensators, electron beam; up to 5 mev (UMLS:HCPCS:G6011); or

Radiation treatment delivery,3 or more separate treatment areas, custom blocking, tangential ports, wedges, rotational beam, compensators, electron beam; 6-10 mev (UMLS:HCPCS:G6012); or

Radiation treatment delivery,3 or more separate treatment areas, custom blocking, tangential ports, wedges, rotational beam, compensators, electron beam; 11-19 mev (UMLS:HCPCS:G6013); or

Radiation treatment delivery,3 or more separate treatment areas, custom blocking, tangential ports, wedges, rotational beam, compensators, electron beam; 20 mev or greater (UMLS:HCPCS:G6014); or

Compensator-based beam modulation treatment delivery of inverse planned treatment using 3 or more high resolution (milled or cast) compensator, convergent beam modulated fields, per treatment session (UMLS:HCPCS:G6016); or

Stereotactic body radiation therapy, treatment delivery, per fraction to 1 or more lesions, including image guidance, entire course not to exceed 5 fractions (UMLS:CPT:77373).

ICI: The terms in this group occurred at any time

Patients must have:

any of the following:

Injection, pembrolizumab, 1 mg (UMLS:HCPCS:J9271); or

Injection, pembrolizumab, 1 mg (deprecated 2015) (UMLS:HCPCS:C9027); or

Injection, nivolumab, 1 mg (UMLS:HCPCS:J9299); or

Injection, nivolumab and relatlimab-rmbw, 3 mg/1 mg (UMLS:HCPCS:J9298); or

Injection, nivolumab, 1 mg (deprecated 2015) (UMLS:HCPCS:C9453); or

Injection, ipilimumab, 1 mg (UMLS:HCPCS:J9228); or
Injection, atezolizumab, 10 mg (UMLS:HCPCS:J9022); or
Injection, atezolizumab, 10 mg (deprecated 2017) (UMLS:HCPCS:C9483); or
Injection, avelumab, 10 mg (UMLS:HCPCS:J9023); or
Injection, avelumab, 10 mg (deprecated 2017) (UMLS:HCPCS:C9491); or
Injection, durvalumab, 10 mg (UMLS:HCPCS:J9173); or
Injection, cemiplimab-rwlc, 1 mg (UMLS:HCPCS:J9119); or
Injection, durvalumab, 10 mg (deprecated 2018) (UMLS:HCPCS:C9492); or
Injection, cemiplimab-rwlc, 1 mg (deprecated 2019) (UMLS:HCPCS:C9044).

CR: Any instance of CR occurred within 6 months and 31 days before any instance of ICI
Patients must have:
any of the following:

Stereotactic radiosurgery (particle beam, gamma ray, or linear accelerator); 1 simple cranial lesion (UMLS:CPT:61796); or
Stereotactic radiosurgery (particle beam, gamma ray, or linear accelerator); 1 complex cranial lesion (UMLS:CPT:61798); or
Stereotactic radiosurgery (particle beam, gamma ray, or linear accelerator); each additional cranial lesion, complex (List separately in addition to code for primary procedure) (UMLS:CPT:61799); or
Application of stereotactic headframe for stereotactic radiosurgery (List separately in addition to code for primary procedure) (UMLS:CPT:61800); or
Radiation treatment delivery, stereotactic radiosurgery (SRS), complete course of treatment of cranial lesion(s) consisting of 1 session; multi-source Cobalt 60 based (UMLS:CPT:77371); or
Radiation treatment delivery, stereotactic radiosurgery (SRS), complete course of treatment of cranial lesion(s) consisting of 1 session; linear accelerator based (UMLS:CPT:77372); or
Stereotactic radiation treatment management of cranial lesion(s) (complete course of treatment consisting of 1 session) (UMLS:CPT:77432); or
Image-guided robotic linear accelerator-based stereotactic radiosurgery, complete course of therapy in one session or first session of fractionated treatment (UMLS:HCPCS:G0339); or
Image-guided robotic linear accelerator-based stereotactic radiosurgery, delivery including collimator changes and custom plugging, fractionated treatment, all lesions, per session, second through fifth sessions, maximum five sessions per course of treatment (UMLS:HCPCS:G0340); or
Radiation treatment delivery, superficial and/or ortho voltage, per day (UMLS:CPT:77401); or
Radiation treatment delivery, >=1 MeV; simple (UMLS:CPT:77402); or
Radiation treatment delivery, >=1 MeV; intermediate (UMLS:CPT:77407); or
Radiation treatment delivery, >=1 MeV; complex (UMLS:CPT:77412); or
Radiation treatment delivery, single treatment area,single port or parallel opposed ports, simple blocks or no blocks: 6-10 mev (UMLS:HCPCS:G6004); or
Radiation treatment delivery, single treatment area,single port or parallel opposed ports, simple blocks or no blocks: 11-19 mev (UMLS:HCPCS:G6005); or
Radiation treatment delivery, single treatment area,single port or parallel opposed ports, simple blocks or no blocks: 20 mev or greater (UMLS:HCPCS:G6006); or
Radiation treatment delivery, 2 separate treatment areas, 3 or more ports on a single treatment area, use of multiple blocks: up to 5 mev (UMLS:HCPCS:G6007); or
Radiation treatment delivery, 2 separate treatment areas, 3 or more ports on a single treatment area, use of multiple blocks: 6-10 mev (UMLS:HCPCS:G6008); or
Radiation treatment delivery, 2 separate treatment areas, 3 or more ports on a single treatment area, use of multiple blocks: 11-19 mev (UMLS:HCPCS:G6009); or
Radiation treatment delivery, 2 separate treatment areas, 3 or more ports on a single treatment area, use of multiple blocks: 20 mev or greater (UMLS:HCPCS:G6010); or
Radiation treatment delivery,3 or more separate treatment areas, custom blocking, tangential ports,

wedges, rotational beam, compensators, electron beam; up to 5 mev (UMLS:HCPCS:G6011); or
Radiation treatment delivery,3 or more separate treatment areas, custom blocking, tangential ports, wedges, rotational beam, compensators, electron beam; 6-10 mev (UMLS:HCPCS:G6012); or
Radiation treatment delivery,3 or more separate treatment areas, custom blocking, tangential ports, wedges, rotational beam, compensators, electron beam; 11-19 mev (UMLS:HCPCS:G6013); or
Radiation treatment delivery,3 or more separate treatment areas, custom blocking, tangential ports, wedges, rotational beam, compensators, electron beam; 20 mev or greater (UMLS:HCPCS:G6014); or
Compensator-based beam modulation treatment delivery of inverse planned treatment using 3 or more high resolution (milled or cast) compensator, convergent beam modulated fields, per treatment session (UMLS:HCPCS:G6016); or
Stereotactic body radiation therapy, treatment delivery, per fraction to 1 or more lesions, including image guidance, entire course not to exceed 5 fractions (UMLS:CPT:77373).

Appendix C – Text Representation of the Outcomes Definition

This analysis includes the following outcomes:

Unnamed Outcome
Patients must have:
any of the following:
Ill-defined and unknown cause of mortality (UMLS:ICD10CM:R99); or
Ill-defined and unknown cause of mortality (R99) (UMLS:ICD10CM:R99-R99); or
Illness, unspecified (UMLS:ICD10CM:R69); or
Deceased (Deceased).
