# Supplementary material for: Survival Outcomes of Immune Checkpoint Inhibitors in Conjunction with Cranial Radiation for Older Adults with Non-Small Cell Lung Cancer and Synchronous Brain Metastasis
Source: Curr Oncol. 2025 Sep 5;32(9):499. doi: 10.3390/curroncol32090499 (PMC12468429; doi:10.3390/curroncol32090499)
Supplement: Supplementary file 1 [file curroncol-32-00499-s001.zip › Table S1-S4-curroncol-3723038-supplementary.pdf]

Supplementary Table S1: Review of retrospective studies involving patients with NSCLC and BM treated with "concurrent" ICI and CR

Author, Year	Country	Primary Cancer	BMs	Design	Radiation Type	Study group	No. of patients	NSCLC specific sample size	Time interval for concurrent	Age of study participants	Overall survival Results
Ahmed, 2017 [37]	USA	NSCLC+BM	Undefined	Retrospective single institution	SRS/FSRT	SRS and ICI	17		≤6 months	Median age 60 years; range (44-79 years)	No significant difference when ICI given during/after SRS compared to before
Chen, 2018 [47]	USA	NSCLC /Melanoma/RCC with BM	Undefined	Retrospective single institution	SRS/SRT	Concurrent SRS-SRT and ICI	28	1	≤2w	Age ≥70 years: 77% - 86%	SRS-SRT with concurrent ICI was associated with improved OS compared with non-concurrent SRS-SRT and ICI
						Nonconcurrent SRS-SRT and ICI	51	35			
						SRS without ICI	181	143			

											(HR: 2.40; p=0.006)
Enright, 2021 [38]	USA	NSCLC+BM	Newly diagnosed	Retrospective single institution	SRS/FSRT	SRT plus ICI	33		≤3m	Median age: 63; range (46-81)	Only univariate analysis. SRT with ICI has better OS than SRT alone [HR: 0.46, 95% CI (0.23-0.91)]
Khan, 2021 [46]	China	NSCLC+BM	Undefined	Retrospective single institution	WBRT	WBRT+anti PD1 (ICI)	10		≤30 days	Mean age: 55.6 years; Std. Dev:12.5	No significant difference between WBRT plus anti-PD1 therapy compared to WBRT alone
						WBRT alone	11				
Koenig, 2019 [39]	USA	Melanoma, NSCLC, RCC, Breast, other	intact/resected	Retrospective single institution	SRS	Concurrent ICI and SRS	70	31	≤4 wks	Median age: 66 years; range (19-90)	Concurrent use of SRS and ICIs was associated

						Non-concurrent ICI SRS	27	51	>4 wks to 5 m		with lower hazard of death compared with non-concurrent SRS and ICI (HR:0.57; 95% CI: 0.33-0.99)
Kotecha, 2019 [48]		Mixed		Retrospective single institution	SRS	Concurrent ICI and SRS therapy	564	99	Received ICI at some point during disease course	Age ≥60 years: 56	No significant difference in response was observed in patients received concurrent ICI compared to non-concurrent.
					SRS	Immediate ICI and SRS therapy	367 of 264		±5 biological half lives		
						SRS alone	143	75			
Lee, 2021 [40]	Korea	NSCLC with BM	SBM: 40; MBM: 37	Retrospective single institution	GKS	ICI with concurrent GKS			≤14 days	Median age: 60 range (42-79)	OS was significantly shorter in

						ICI with non-concurrent GKS					ICI alone compared to ICI with non-concurrent GKS
						ICI alone					
Qian, 2020 [41]	USA	NSCLC, Melanoma with BM	Undefined	Retrospective single institution	SRS/FSRT or WBRT		110	35	≤90 days	Median age: 61; range (53-69)	Not assessed
Schapira, 2018 [42]		NSCLC+BM	Undefined	Retrospective single institution	SRS	SRS concurrent	8		<1 m	Median age: 63; range (42-84)	OS was significantly improved among patients receiving concurrent SRS and ICI compared with SRS before or after ICI
						SRS before	24				
						SRS after	5				
Shepard, 2019 [43]	USA	NSCLC	Undefined	Retrospective single institution	SRS	Concurrent ICI	17		≤3m	Mean age: 64 years	No significant difference in overall survival between concurren
						ICI naïve	34				

											t ICI and ICI naïve cohorts
Singh, 2019 [44]	USA	NSCLC+BM	Undefined	Retrospective single institution	SRS	ICI with SRS	39		undefined	Median age: 61.9 years; range (28-87.5)	No significant difference in OS was seen between study groups
						Chemotherapy with SRS	46				
Yomo, 2023 [45]	Japan	lung+BM (NSCLC, SCLC)	Untreated	Retrospective single institution	SRS	ICI+SRS	89		≤3 m	Median age: 69; range (62-75)	Among NSCLC subgroup, ICI+SRS had lower hazard of death compared to SRS alone (HR: 0.63; 95%CI: 0.44-0.89)
						SRS alone	89				

Abbreviations: USA: United States of America; NSCLC: Non-small cell lung cancer; BM: Brain Metastasis; SRS: Stereotactic radiosurgery; ICI: Immune checkpoint inhibitors; FSRT: Fractionated stereotactic radiotherapy; RCC: Renal cell carcinoma; SRT: Superficial radiation therapy; OS: Overall survival; HR: Hazard ratio; WBRT: Whole brain radiation therapy; MF-SRT: Multifraction stereotactic radiotherapy; SBM: Synchronous brain metastasis; MBM: Metachronous brain metastasis; GKS: Gamma knife surgery

Supplementary Table S2: Adjusted hazard ratios of overall survival from multivariate cox proportional hazard regression analysis with change point of 90 and 300 days. Comparing within 15 days and more than 15 days intervals

Variables	OS HR (95% C.I)	p-value
**Subsequent ICI-CR treatment received**		
Within 15 days vs. more than 15 days before 90 days change point	0.69 (0.41-1.15)	0.1625
Within 15 days vs. more than 15 days between 90-300 days change point	2.09 (1.13-3.87)	**0.0177**
Within 15 days vs. more than 15 days after 300 days change point	0.39 (0.18-0.84)	**0.0171**
**Age at diagnosis**	1.02 (0.99-1.05)	0.1808
**Marital status at diagnosis**		
Not married	-	-
Married	0.89 (0.63-1.27)	0.5537
**Sex**		
Female	-	-
Male	1.17 (0.83-1.63)	0.3557
**Race/Ethnicity**		
Non-Hispanic White	-	-
Other	0.91 (0.56-1.49)	0.7265
**Census tract poverty indicator**		
0%-<5%	-	-

5-<10	0.83 (0.54-1.27)	0.4079
10-<20	0.64 (0.41-1.00)	0.0532
20-100	0.98 (0.57-1.68)	0.9471
unknown	0.64 (0.29-1.41)	0.2717
**Rurality of patient's county of residence**		
Metropolitan area	-	-
Non-metropolitan area	1.15 (0.68-1.93)	0.5976
**Metastases at diagnosis**		
Bone metastasis	0.93 (0.64-1.35)	0.7230
Liver metastasis	0.89 (0.57-1.40)	0.6371
Lung metastasis	1.18 (0.80-1.75)	0.3837
**Charlson comorbidity index score**		
0	-	-
1	0.81 (0.54-1.19)	0.2911
≥2	0.75 (0.49-1.14)	0.1832
**Baseline ECOG performance status proxy**		
0-2	-	-
3-4	0.83 (0.27-2.51)	0.7432
**Histology**		
Adenocarcinoma	-	-
Squamous cell	1.15 (0.75-1.76)	0.5031
Other type	0.83 (0.54-1.28)	0.4133
**Tumor grade**		
1-2	-	-
3-4	0.60 (0.36-1.23)	0.1973
Undetermined	0.64 (0.35-1.15)	0.1384
**Treatments within 6 months from diagnosis**		
SRS	1.02 (0.73-1.41)	0.8992
Chemotherapy	1.07 (0.74-1.55)	0.7014
Neurosurgical resection	1.08 (0.67-1.74)	0.7353

Abbreviations: OS: Overall Survival; HR: Hazard Ratio, CI: Confidence Intervals, ICI: Immune Checkpoint Inhibitors, CR: Cranial Radiation, ECOG PS: Eastern Cooperative Oncology Group Performance Status, SRS: Stereotactic Radiosurgery

All p-values significant at $\alpha \leq 0.05$

Supplementary Table S3: Adjusted hazard ratios of overall survival of subgroup analysis of patients with ICI and SRS from multivariate cox proportional hazard regression analysis

Variables	OS HR (95% C.I)	p-value
**Subsequent ICI-SRS treatment received**		
More than 30 days	-	-
Within 15 days vs.	1.30 (0.61-2.81)	0.4896
16 to 30 days	2.71 (0.87-8.38)	0.0830
Within 15 days vs. 16 to 30 days	0.43 (0.20-1.15)	0.1028
**Age at diagnosis**	1.05 (1.00-1.11)	**0.0187**
**Marital status at diagnosis**		
Not married	-	-
Married	1.08 (0.59-1.96)	0.7977
**Sex**		
Female	-	-
Male	0.83 (0.47-1.48)	0.5425
**Race/Ethnicity**		
Non-Hispanic White	-	-
Other	1.20 (0.55-2.59)	0.6407
**Census tract poverty indicator**		

0%-<5%	-	-
5-<10	1.35 (0.66-2.78)	0.4036
10-<20	1.15 (0.58-2.27)	0.6801
20-100	1.22 (0.45-3.34)	0.6885
unknown	0.55 (0.11-2.58)	0.4511
**Rurality of patient's county of residence**		
Metropolitan area	-	-
Non-metropolitan area	0.76 (0.27-2.13)	0.6080
**Metastases at diagnosis**		
Bone metastasis	1.44 (0.82-2.52)	0.1939
Liver metastasis	1.17 (0.58-2.36)	0.6477
Lung metastasis	1.55 (0.82-2.93)	0.1748
**Charlson comorbidity index score**		
0	-	-
1	0.95 (0.46-1.95)	0.8943
≥2	1.07 (0.55-2.08)	0.8222
**Baseline ECOG performance status proxy**		
0-2	-	-
3-4	0.30 (0.06-1.50)	0.1438
**Histology**		
Adenocarcinoma	-	-
Squamous cell	0.59 (0.26-1.32)	0.2032
Other type	2.56 (1.21-5.44)	**0.0141**
**Tumor grade**		
1-2	-	-
3-4	0.62 (0.22-1.72)	0.3679
Undetermined	0.86 (0.35-2.11)	0.7472
**Treatments within 6 months from diagnosis**		
Chemotherapy	2.45 (1.24-4.85)	**0.0096**
Neurosurgical resection	0.77 (0.36-1.62)	0.4930

Abbreviations: OS: Overall Survival; HR: Hazard Ratio, CI: Confidence Intervals, ICI: Immune Checkpoint Inhibitors, CR: Cranial Radiation, ECOG PS: Eastern Cooperative Oncology Group Performance Status, SRS: Stereotactic Radiosurgery

All p-values significant at $\alpha \leq 0.05$

Supplementary Table S4: Adjusted hazard ratios of overall survival of subgroup analysis of patients with ICI and non-SRS from multivariate cox proportional hazard regression analysis

Variables	OS HR (95% C.I)	p-value
**Subsequent ICI-non-SRS treatment received**		
More than 30 days	-	-
Within 15 days vs.	0.94 (0.53-1.67)	0.8489
16 to 30 days	1.07 (0.54-2.09)	0.8404
Within 15 days vs. 16 to 30 days	0.88 (0.47-1.64)	0.7984
**Age at diagnosis**	1.03 (0.99-1.07)	0.1440
**Marital status at diagnosis**		
Not married	-	-
Married	1.13 (0.73-1.74)	0.5661
**Sex**		
Female	-	-
Male	0.52 (0.33-1.23)	0.0078
**Race/Ethnicity**		
Non-Hispanic White	-	-
Other	1.63 (0.32-2.59)	0.1789
**Census tract poverty indicator**		
0%-<5%	-	-
5-<10	0.98 (0.51-1.90)	0.9743
10-<20	0.81 (0.43-1.54)	0.5361
20-100	2.07 (1.01-4.24)	0.0455
unknown	1.46 (0.62-3.45)	0.3778
**Rurality of patient's county of residence**		
Metropolitan area	-	-
Non-metropolitan area	1.12 (0.56-2.24)	0.7366
**Metastases at diagnosis**		
Bone metastasis	1.39 (0.85-2.29)	0.1855
Liver metastasis	1.06 (0.58-1.95)	0.8326

Lung metastasis	1.71 (1.02-2.87)	0.0400
**Charlson comorbidity index score**		
0	-	-
1	0.76 (0.44-1.32)	0.3437
≥2	0.54 (0.30-0.98)	0.0460
**Baseline ECOG performance status proxy**		
0-2	-	-
3-4	4.46(0.94-20.96)	0.0583
**Histology**		
Adenocarcinoma	-	-
Squamous cell	1.36 (0.69-2.68)	0.3646
Other type	1.03 (0.59-1.78)	0.9082
**Tumor grade**		
1-2	-	-
3-4	1.50 (0.60-3.73)	0.3827
Undetermined	2.79 (1.10-7.02)	0.0294
**Treatments within 6 months from diagnosis**		
Chemotherapy	0.78 (0.45-1.34)	0.3826
Neurosurgical resection	0.49 (0.26-0.93)	0.0308

Abbreviations: OS: Overall Survival; HR: Hazard Ratio, CI: Confidence Intervals, ICI: Immune Checkpoint Inhibitors, CR: Cranial Radiation, ECOG PS: Eastern Cooperative Oncology Group Performance Status, SRS: Stereotactic Radiosurgery
All p-values significant at $\alpha \leq 0.05$
