# Supplementary figures and images for: Survival Outcomes of Immune Checkpoint Inhibitors in Conjunction with Cranial Radiation for Older Adults with Non-Small Cell Lung Cancer and Synchronous Brain Metastasis
Source: Curr Oncol. 2025 Sep 5;32(9):499. doi: 10.3390/curroncol32090499 (PMC12468429; doi:10.3390/curroncol32090499)

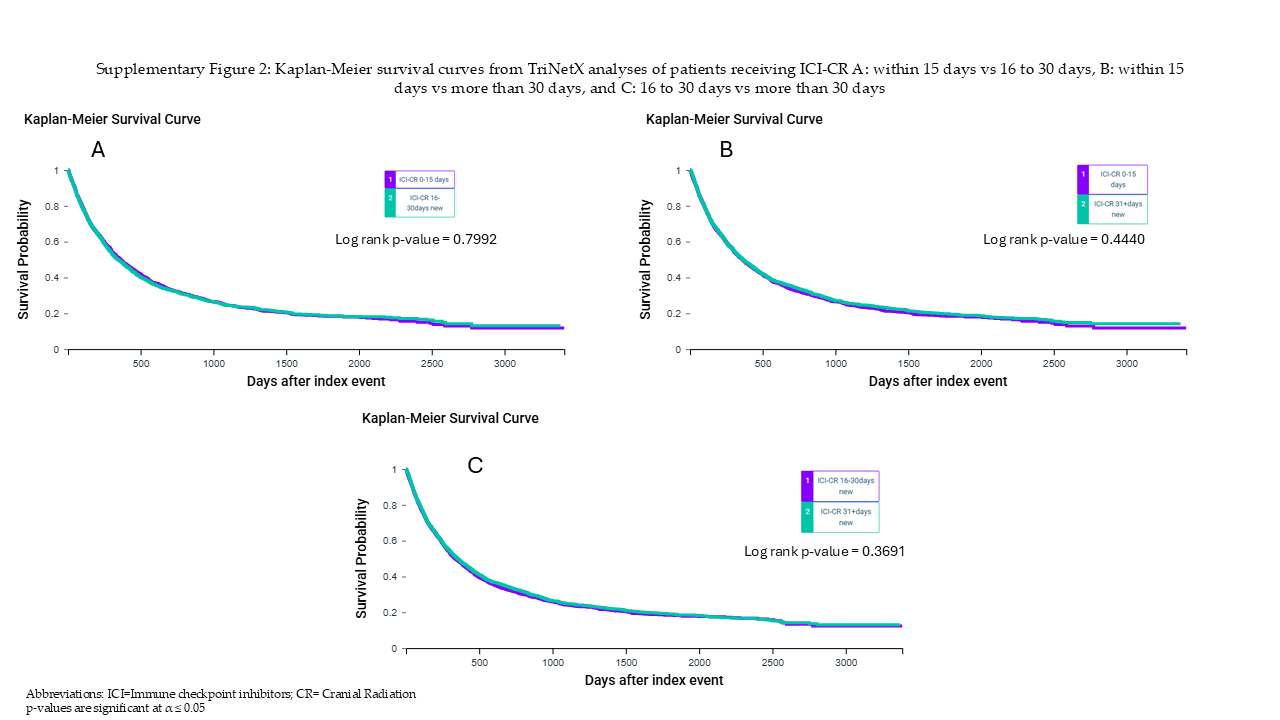

Supplement: Supplementary file 1 [file curroncol-32-00499-s001.zip › Figure S2-curroncol-3723038-supplementary.png]

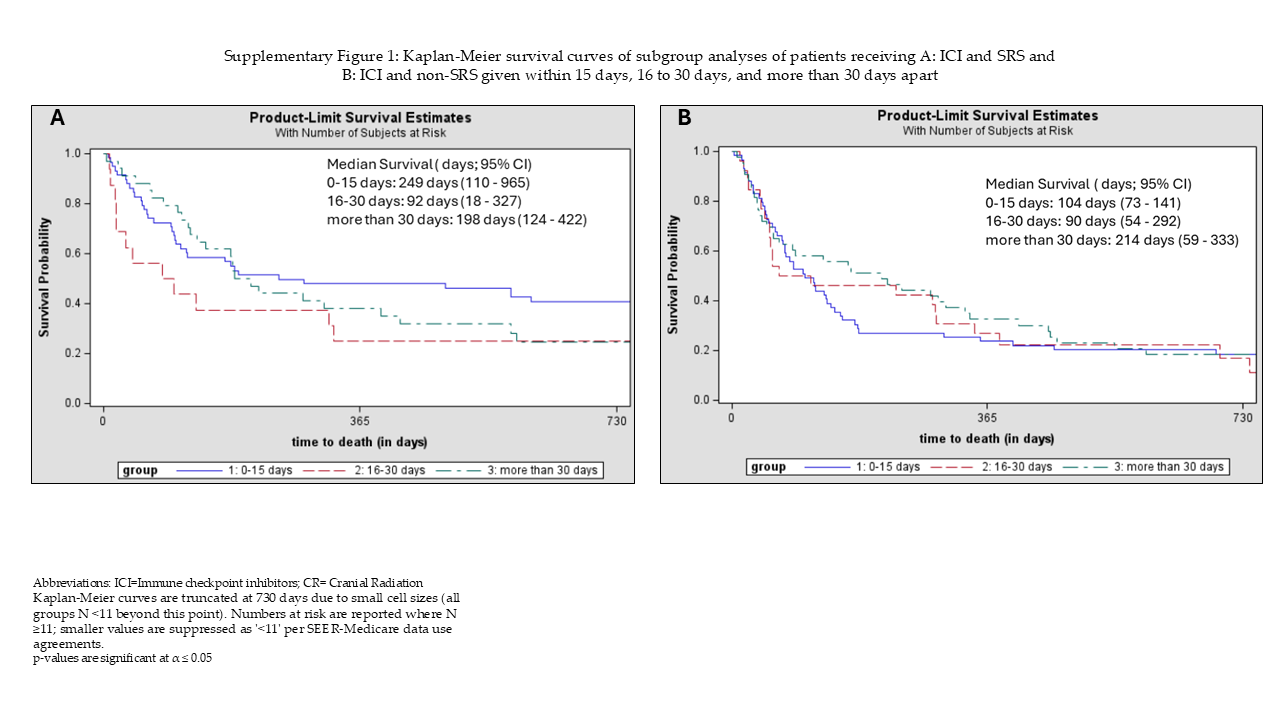

Supplement: Supplementary file 1 [file curroncol-32-00499-s001.zip › Figure S1-curroncol-3723038-supplementary.png]
